# Critical Solvation Structures Arrested Active Molecules for Reversible Zn Electrochemistry

**DOI:** 10.1007/s40820-024-01361-0

**Published:** 2024-03-05

**Authors:** Junjie Zheng, Bao Zhang, Xin Chen, Wenyu Hao, Jia Yao, Jingying Li, Yi Gan, Xiaofang Wang, Xingtai Liu, Ziang Wu, Youwei Liu, Lin Lv, Li Tao, Pei Liang, Xiao Ji, Hao Wang, Houzhao Wan

**Affiliations:** 1 Hubei Yangtze Memory Laboratories, Wuhan, 430205 People’s Republic of China; 2 Hubei Key Laboratory of Micro-Nanoelectronic Materials and Devices, School of Microelectronics, Hubei University, Wuhan, 430062 People’s Republic of China; 3 School of Physical and Mathematical Sciences, Nanyang Technological University, Singapore, 637371 Singapore; 4 School of Optical and Electronic Information, Huazhong University of Science and Technology, Wuhan, 430074 People’s Republic of China; 5 Institute of Optoelectronics Technology, China Jiliang University, Hangzhou, 310018 People’s Republic of China

**Keywords:** Zinc-ion battery, Critical solvation, Helmholtz layer, Arrest active molecule, Reversible zinc anode

## Abstract

Aqueous Zn-ion batteries (AZIBs) have attracted increasing attention in next-generation energy storage systems due to their high safety and economic. Unfortunately, the side reactions, dendrites and hydrogen evolution effects at the zinc anode interface in aqueous electrolytes seriously hinder the application of aqueous zinc-ion batteries. Here, we report a critical solvation strategy to achieve reversible zinc electrochemistry by introducing a small polar molecule acetonitrile to form a “catcher” to arrest active molecules (bound water molecules). The stable solvation structure of [Zn(H_2_O)_6_]^2+^ is capable of maintaining and completely inhibiting free water molecules. When [Zn(H_2_O)_6_]^2+^ is partially desolvated in the Helmholtz outer layer, the separated active molecules will be arrested by the “catcher” formed by the strong hydrogen bond N–H bond, ensuring the stable desolvation of Zn^2+^. The Zn||Zn symmetric battery can stably cycle for 2250 h at 1 mAh cm^−2^, Zn||V_6_O_13_ full battery achieved a capacity retention rate of 99.2% after 10,000 cycles at 10 A g^−1^. This paper proposes a novel critical solvation strategy that paves the route for the construction of high-performance AZIBs.

**Supplementary Information:**

The online version contains supplementary material available at 10.1007/s40820-024-01361-0.

## Highlights

- Critical solvation structure changes the hydrogen bond network through “catchers”.
- Catcher further arrests the active molecules to promote Zn^2+^ deposition.
- The Zn||Zn symmetric battery can stably cycle for 2250 h. Zn||V_6_O_13_ full battery achieved a capacity retention rate of 99.2% after 10,000 cycles.

**Supplementary Information:**

The online version contains supplementary material available at 10.1007/s40820-024-01361-0.

## Graphical abstract

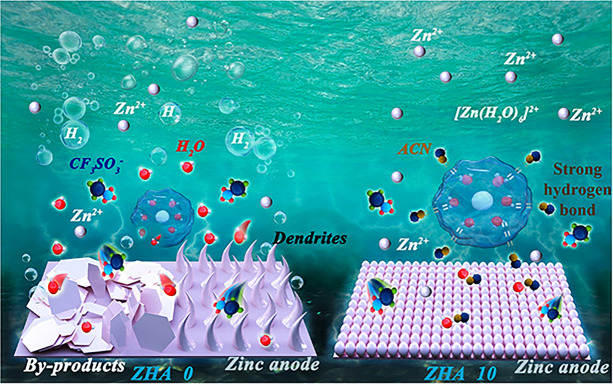

## Introduction

The urgent global demand for energy is promoting the efficient and rapid iterative development of energy storage systems [1–3]. However, with the reports of some battery spontaneous combustion incidents in recent years, the safety of new energy systems has become a hot topic [4–8]. Compared with limited lithium resources and flammable organic electrolytes, safe and efficient (theoretical capacity 820 mAh g^−1^, redox potential − 0.76 V vs. SHE) and resource-abundant aqueous AZIBs are extremely promising to replace Li-ion batteries in the large-scale energy storage applications [9–12]. However, the inherent activity of the Zn anode also causes serious problems of hydrogen evolution, corrosion and dendrite growth [13–16]. The reason for its production can be attributed to two aspects: (1) because [Zn(H_2_O)_6_]^2+^ in the process of desolvation, H_2_O molecules are more inclined to receive electrons, resulting in the hydrogen evolution effect [17–19]. In addition, excessive accumulation of OH^−^ can also lead to corrosion of Zn anode [20, 21]. (2) Unevenly distributed electric field. On the uneven zinc surface, Zn^2+^ will first choose the preferential nucleation site to gather to form the initial zinc dendrites, and then the electroplated Zn^2+^ tends to deposit again at the first dendrite protrusions during the charging process. This disordered two-dimensional deposition phenomenon is called the “tip effect” [22, 23]. These problems will lead to extremely low zinc anode utilization and seriously affect battery performance. Interface modification is an effective means to solve these problems, availably guiding ion/electron transport and altering the distribution of the electric field [24–27]. However, considering the preparation process, durability and scalability of the interface layer, this method will restrict the future large-scale production of zinc-ion batteries.

The optimized solvation structure can effectively inhibit the activity of H_2_O, thereby inhibiting hydrogen evolution and side reactions [28–31]. Furthermore, by inducing the uniform deposition of Zn^2+^, the uneven nature of the internal electric field is also changed, enabling reversible deposition and stripping of Zn^2+^ [32]. Until now, many strategies have been reported to regulate the electrolyte solvation structure [33, 34]. For example, optimizing the solvation structure by developing highly concentrated electrolytes. Lu et al. mentioned that they increased the maximum solubility of electrolyte to 23 m Zn(Ac)_2_ and choose the low-cost Zn(Ac)_2_ to suppress the side reaction (HER) [35]. However, the higher viscosity of the high-concentration electrolyte also reduces the transport kinetics of Zn^2+^. In order to solve this problem, the researchers gradually shifted their attention to organic solvents that can be dissolved with electrolyte salts, by adding suitable organic solvents to rebuild the hydrogen bond network in the electrolyte and regulate the solvation structure of Zn^2+^. For example, Wang et al. [36] proposed to introduce N,N-dimethylacetamide (DMF) as an electrolyte additive to achieve reversible zinc electrochemistry; Mai et al. proposed to construct a deep eutectic solvent of sulfolane-H_2_O to facilitate the stable plating/stripping of Zn^2+^ [37]. What we need to note is that everyone introduces a large number of organic molecules to replace the cationic solvation sheath, which led to the readjustment of the Zn^2+^ solvation sheath. However, the introduction of organic solvents into electrolytes brings about two known solvation effects. One way is for H_2_O to interact with the electrolyte, forming a covalent bond. This helps adjust the hydrogen bond structure in the aqueous-electrolyte, limiting the activity of H_2_O and protecting the zinc anode. The other is that organic substances are inserted into the inner solvation sheath of hydrated zinc ions, replacing the position of H_2_O, and reconstructing the solvation sheath structure of Zn^2+^. However, excessive additives will severely limit the ion transmission efficiency and sacrifice battery performance.

In this paper, we propose a novel optimal solvation effect—critical solvation structure by introducing different proportions of ACN into 1 m Zn(CF_3_SO_3_)_2_-H_2_O electrolyte. We found that in the critical solvation structure, the combination of N–H bonds changes the hydrogen bonding network in water, limiting the free water. In addition, the desorbed active molecules can be “catcher” and arrested during the desolvation process to promote Zn^2+^ deposition. The symmetrical battery can stably cycle for 2250 h under the condition of 1 mAh cm^−2^. The half-cell can maintain a high coulombic efficiency of 99.4% after 10,000 cycles. The assembled Zn||V_6_O_13_ full battery can achieve a capacity retention rate of 99.2% after 10,000 cycles at 10 A g^−1^. This study provides a new idea for the selection of the dosage of organic molecular additives, and provides a new strategy for designing economic and high-efficiency aqueous electrolytes.

## Experimental Section

### Materials

Zn foil (purity 99.99%) and copper foil (purity 99.99%); analytical pure vanadium pentoxide (V_2_O_5_), absolute ethanol (C_2_H_5_OH) and zinc trifluoromethanesulfonate (Zn(CF_3_SO_3_)_2_); anhydrous acetonitrile (purity ≥ 99.5%) are all from Sinopharm Group.

### Preparation of Electrolyte Solution

For Zn(CF_3_SO_3_)_2_-H_2_O, dissolve Zn(CF_3_SO_3_)_2_ powder in deionized (DI) water. The concentration of Zn(CF_3_SO_3_)_2_ in the solution is 1 m. Then, the prepared 1 m Zn(CF_3_SO_3_)_2_ solution and anhydrous acetonitrile (ACN) were mixed according to the mass fraction ratio. The mass fraction proportions of acetonitrile are 0%, 5%, 8%, 10%, 20%, 30%, 50%, and 70%. The mixed electrolyte is recorded as ZHA X (X is the ratio). HA X is a mixture of deionized water and anhydrous acetonitrile in proportion to its mass fraction.

### Preparation of Cathode

First, 1.8 g of V_2_O_5_ was weighed into 20 mL of absolute ethanol and 50 mL of deionized (DI) water, mixed for 1 h, then transferred to a 100 mL reactor, and then reacted in a dry box at 180 °C for 12 h. After cooling to room temperature, remove and filter, wash repeatedly, and dry. Finally, V_6_O_13_ powder was obtained. The prepared V_6_O_13_ powder sample was spread on a stainless-steel mesh using the smear method and sliced into battery pole pieces with a diameter of 1.2 mm for battery packaging. During the smearing process, mix in an agate mortar in a ratio of 7:2:1 (V_6_O_13_ powder: acetylene black: PVDF), and then drop N-methylpyrrolidone (NMP) solution until a viscous mixture. Coated on the treated stainless-steel mesh. The active mass of the final cathode material should be controlled in the 1–2 mg range.

### Characterization of Materials

X-ray diffractometry (XRD) is used to analyze the crystal structure of materials. This paper uses a Bruker D8 Advance X-ray diffractometer. The diffraction angle is selected from 5° to 80°. The JSM-7100F field emission scanning electron microscopy (SEM) equipment produced by JEOL Company of Japan was used to characterize the microstructure of the material by SEM, and the detailed characteristics under different magnification were observed. The acceleration voltage is 15 kV and the test distance is 10 mm. The X-ray photoelectron spectrometer (XPS) used is ESCALAB 250Xi model of Thermo SCIENTIFIC Company. The voltage and current of the X-ray tube were set to 15 kV and 10 mA. Renishao's Raman spectrometer was selected to analyze the materials and electrolyte. The laser wavelength is 532 nm. The fourier transform infrared spectrometer (FT-IR) was selected from the American Thermo Fisher infrared spectrometer, and the selected spectral range was 400–4000 cm^−1^. The optical microscope (MXFMS-BD) is used to monitor the dynamic change process of the material surface in real time (this paper uses it to observe the dynamic change of the cross section of zinc during the stripping/deposition process). It is equipped with a constant current battery tester to meet the charging and discharging conditions.

### Electrochemical Measurements

The cyclic voltammetry (CV), linear sweep voltammetry (LSV), tafel curve, response current curve (CA) and electrochemical AC impedance spectroscopy (EIS) curves of Zn||Zn symmetric batteries and Zn||Cu and Zn||Ti half batteries were tested by CHI 760E electrochemical workstation. The cycle life, rate performance, coulomb efficiency and corresponding polarization curves of Zn||Zn batteries were measured by an eight-channel tester (Xinwei BEWARE).

The relevant tests of CV, galvanostatic charge–discharge (GCD), charge and discharge cycle time and rate were carried out on the Zn||V_6_O_13_ full battery. The specific capacity *C* (mAh g^−1^), power density *P* (Wh kg^−1^) and energy density *E* (kW kg^−1^) of the packaged zinc-ion battery can be calculated by the following formula:

1 $$ C = \frac{{{\text{i}}\Delta {\text{t}}}}{{{3}{\text{.6}}\;{\text{m}}}} $$

2 $$ E = \frac{1000}{{3600}}C\Delta V $$

3 $$ P = \frac{3600}{{1000}}*\frac{E}{\Delta t} $$

Among them, Δ*t* is the discharge time (s), *I* is the discharge current (A), *m* is the active mass (g), and Δ*V* is the voltage difference (V).

### MD Simulation and DFT Computations

Classical molecular dynamics simulations (MD) were conducted using the LAMMPS [38] software package. The initial periodic systems were constructed using PACKMOL [39] and Moltemplate (http://www.moltemplate.org/). The properties of H_2_O were assessed using the tip3p parameters. The force field parameters for Zn^2+^ and OTf^−^ ions were adopted from previous publications [40, 41], including partial charges. The force field parameters for acetonitrile (ACN) were derived from the OPLS-AA parameters [42]. The velocity-Verlet algorithm was utilized to numerically integrate the equations of motion, employing a time step of 1 fs. The simulation protocol consisted of the following steps:

1. Langevin dynamics were carried out at 500 K for 1 ns.
2. NPT runs at 298 K were performed for 5 ns to ensure equilibrium salt dissociation.
3. Subsequently, NVT runs were conducted for 10 ns at 298 K.

The solvation structures of the electrolyte were extracted from the last 5 ns of the trajectory. Visualization of the electrolyte structures was accomplished using VESTA [43] and VMD [44].

Quantum chemistry calculations were conducted using the ORCA quantum chemical program [45] and Multiwfn [46] to predict solvent binding energies. The B3LYP-D3/6-311+G** level was employed to optimize molecular geometries, while the SMD implicit solvation model and M06-2X/jun-cc-pVTZ level were used to calculate binding energies.

## Results and Discussion

### Critical Solvation Characterization

**Fig. 1:**
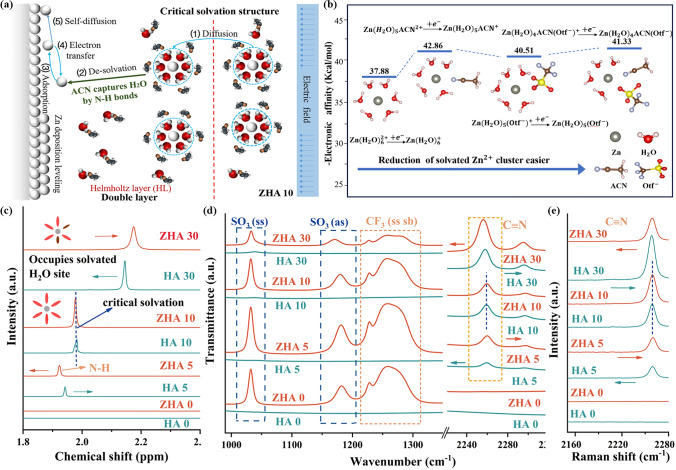
**a** Electroplating mechanism of Zn^2+^ in ZHA 10 electrolytes. **b** Electron affinity comparison of different solvated sheaths. **c** Comparison of N–H bond NMR spectra in different electrolytes. **d** Comparison of infrared spectra in different electrolytes. **e** Comparison of Raman spectra of C≡N bonds in different electrolytes

The critical solvation structure proposed in this paper is based on the organic molecule acetonitrile with low polarity and high dielectric constant (*ε* = 35.9) that combines with water through N–H bonds to reconstruct a hydrogen bond network [47–50]. First, different mass fractions of ACN were dissolved in 1 m Zn(CF_3_SO_3_)_2_ to form ZHA X (X = 0, 5, 8, 10, 20, 30, 50, 70) electrolyte to explore the priority and influence of two different solvation effects. When the electrolyte is ZHA 0, [Zn(H_2_O)_6_]^2+^ is formed in the outer Helmholtz layer (OHL) of the electric double layer by self-diffusion [51, 52]. Then during the desolvation process, the desorbed active molecules can accept electrons from the surface of the zinc anode, and then a HER occurs to produce H_2_ [53]. The remaining OH^–^ will generate Zn(OH)_2_ by-products on the surface of Zn anode [54]. The hydrogen evolution effect and the Zn^2+^ deposition effect is a pair of competing effects. Hydrogen evolution will reduce the reversibility of Zn^2+^ deposition/stripping, resulting in a zinc-poor state and causing corrosion [16, 55, 56]. The generated H_2_ will also be adsorbed on the Zn anode to affect the nucleation process of Zn (Fig. S1). As shown in Fig. 1a, when the electrolyte is ZHA 10, The critical solvation structure formed suppresses free water in the electrolyte. During the subsequent desolvation process, the "catcher" can also arrest the detached active molecules, thereby inhibiting the corrosion of HER and Zn anodes. When the electrolyte is ZHA > 10, ACN will enter the solvation structure of Zn^2+^ and replace the original position of H_2_O (Fig. S2). However, this solvated structure is unstable and easy to self-decompose. Moreover, since the polarity of ACN is weaker than that of H_2_O, the contact angle between the electrolyte and the Zn anode becomes smaller and smaller after the addition of ACN (Fig. S3). At this time, excess ACN will be preferentially adsorbed on the Zn anode, thus affecting the deposition of Zn^2+^. Excess ACN can significantly affect the viscosity and ion transport efficiency of the electrolyte (Fig. S4). As shown in Fig. 1b, we compared the electron affinity relationship between Zn(H_2_O)_6_^2+^ and the different solvated structure. Zn(H_2_O)_6_^2+^ is lower than Zn(H_2_O)_5_(ACN)^2+^, Zn(H_2_O)_5_(Otf^−^)^+^, Zn(H_2_O)_4_(ACN)(Otf^−^)^+^. It shows that when ACN participates in the remodeling of the solvation sheath of Zn^2+^, the overall stability of the electrolyte is reduced. Further reveal the differences in the stability of different solvated structures.

Spectral analysis further shows that in different solvation structures, N–H bonds are preferentially formed with water in the electrolyte, changing the hydrogen bond network. Figure S5 tests the electrolyte magnetic resonance spectra of different solvation structures 1 m Zn(CF_3_SO_3_)_2_-H_2_O-ACN (ZHA) and H_2_O-ACN (HA). The N–H bond peak is generated, indicating that the hydrogen bond network of water is changed. As shown in Fig. 1c, when the amount of ACN gradually increased, the peak of N–H also gradually shifted to the high field, proving the increase of N–H bond. In 1 m Zn(CF_3_SO_3_)_2_-H_2_O, the overall N–H peak also gradually moves to the high field, which is consistent with changes in H_2_O. The peak position of N–H is the same in ZHA 10 and HA 10 electrolytes. When the addition ratio increases to 30%, the N–H peak in 1 m Zn(CF_3_SO_3_)_2_ shifts to a higher field compared with that in water. It is proved that when the addition ratio is greater than 10%, ACN participates in the solvation structure of Zn^2+^, so the peak of N–H shifts to the high field. Therefore, in the critical solvation structure (ZHA 10), the formation of N–H bonds can maximize the suppression of water activity without destroying the solvation structure of Zn^2+^, leaving the [Zn(H_2_O)_6_]^2+^ solvation sheath structures in a stable state.

In the Fourier-transformed infrared spectra (FTIR) spectrum of Fig. 1d, the peak of the C≡N triple bond stretching vibration was observed at 2260 cm^−1^. The symmetric stretch (ss) peak of SO_3_ around 1031 cm^−1^ and the antisymmetric stretch (as) peak at 1181 cm^−1^ can be observed in a solution of 1 m Zn(CF_3_SO_3_)_2_; the peaks of symmetric stretching (ss) and symmetric bending (sb) of CF_3_ within 1200–1300 cm^−1^ [57]. When more ACN is added, the C≡N bonds in the HA electrolyte begin to move toward the lower band (red shift) because of the formation of N–H bonds, which weakens the strength of the C≡N bonds. Correspondingly, in the ZHA electrolyte, it also conforms to this rule. When the addition ratio is 10%, the C≡N peaks of HA 10 and ZHA 10 electrolyte solutions are at the same position. When the addition ratio is greater than 10%, the peak red shift of C≡N is more obvious in ZHA 30 electrolyte. It shows that at this time, ACN not only has a hydrogen bond with H_2_O, but also starts to replace H_2_O in [Zn(H_2_O)_6_]^2+^, and starts the solvation structure reorganization process of Zn^2+^. In the Raman spectrum of Fig. S6, the peaks of C–S and S=O in the solution of 1 m Zn(CF_3_SO_3_)_2_ and C–C and C≡N in ACN can be observed. The change rule of C≡N described in Fig. 1e is also consistent with the results of infrared spectroscopy and NMR. Further, we also tested the pH values of different electrolytes (Fig. S7) and the corresponding physical parameters (Fig. S8). The comparison results show that we successfully confirmed this novel solvation state-the critical solvation structure.

### Critical Solvation Mechanism

**Fig. 2:**
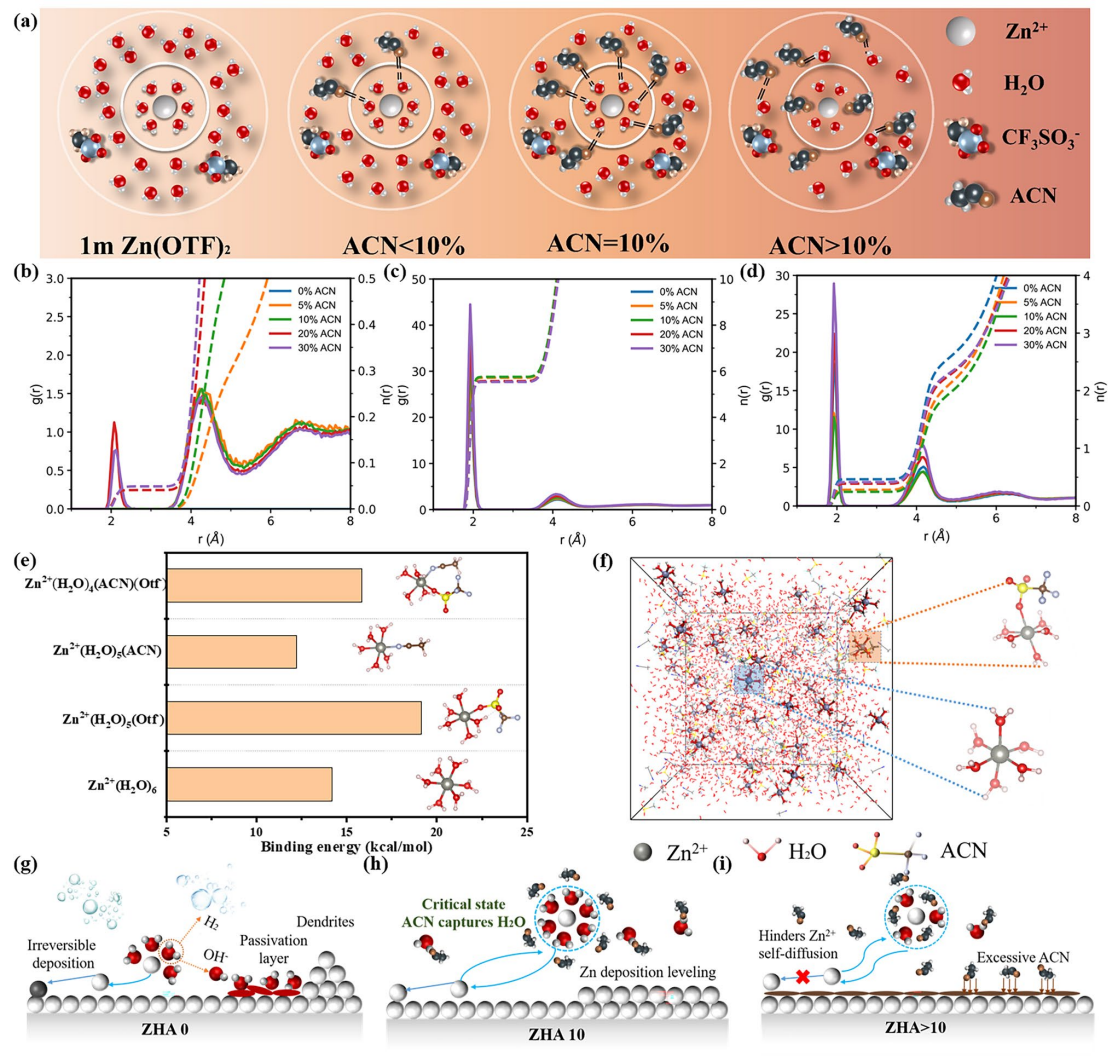
**a** Solvation structure change process in 1 m Zn(CF_3_SO_3_)_2_-H_2_O-ACN_x_ electrolyte. The solvation effect of different mass fractions of ACN in **b** ACN, **c** H_2_O, and **d** Zn(CF_3_SO_3_)_2_ solvent. **e** Comparison binding energy of 1 m Zn(CF_3_SO_3_)_2_-H_2_O-ACN in different states. **f** Solvation mechanism. Zn^2+^ deposition changes in **g** ZHA 0, **h** ZHA 10, and **i** ZHA > 10 electrolytes

Based on the above experimental results, Fig. 2a describes the solvation structure change process in 1 m Zn(CF_3_SO_3_)_2_ solution. First, in the 1 m Zn(CF_3_SO_3_)_2_ electrolyte, [Zn(H_2_O)_6_]^2+^ dominates the solvation structure. However, the H_2_O detached by [Zn(H_2_O)_6_]^2+^ in this state in the desolvation sheath is extremely active, which will lead to various by-products and enhanced hydrogen evolution effects during the cycle. After the introduction of ACN, it first forms a hydrogen bond structure with free water, which helps the electrolyte limit the freedom of H_2_O. When the critical solvation state is reached. It not only stabilizes the structure of [Zn(H_2_O)_6_]^2+^, but also arrests the detached active molecules through a "catcher" during desolvation. When ACN > 10%, ACN is gradually inserted into the Zn^2+^ solvation sheath, replacing the original H_2_O position, which also causes the original stable H_2_O to be squeezed out and become active and loose water molecules again.

Furthermore, different solvated structural forms were calculated by molecular dynamics simulations. The solvation structure of the electrolyte was quantitatively studied by radial distribution function (RDF, g(r), solid line) and integral partition number (ICN, n(r), dashed line) [58]. Figure 2b illustrates the radial distribution functions and coordination numbers of zinc ions and ACN in electrolytes with different ACN concentrations. It can be observed that ZHA 10 is at a critical concentration. Beyond 10%, acetonitrile starts to enter the first solvation shell of zinc ions, replacing water molecules in the solvation structure of zinc ions. Figure 2c shows the radial distribution functions and coordination numbers of zinc ions and water for different ACN concentrations. Combined with Fig. 2b, it is found that ZHA 10, compared to higher ACN concentrations (20/30), has more zinc ions coordinated with water, making it easier to maintain the stable structure of hydrated zinc ions. Figure 2d presents the radial distribution functions and coordination numbers of zinc ions and anions for different ACN concentrations. It is observed that, compared to other additive ratios, ZHA 10 has the least coordination between zinc ions and anions. The specific coordination number values are shown in Table S1. In other words, ZHA 10 is at a critical solvation structure, altering only the hydrogen bonding network of water without disrupting the structure of hydrated zinc ions. During the Zn^2+^ deposition process, the "catcher" can arrest the detached active molecules to ensure that Zn^2+^ can be deposited evenly and neatly. The binding energies of H_2_O, ACN, Otf^−^ and Zn^2+^ were calculated using density functional theory, and the binding energy of ACN is weaker than that of H_2_O (Fig. 2e). Therefore, when the addition ratio is low, it will not participate in the Zn^2+^ solvation structure. Figure 2f shows the solvation mechanism from the molecular dynamic simulation. Through electron affinity, it can be found that when ACN enters the solvation sheath of Zn^2+^ (ACN > 10%), the electron affinity of these three types is reduced. The stability of solvation structure is deteriorated. The spectra and the related theoretical calculations together indicate the establishment of the critical solvation structure theory. In ZHA 0 electrolyte, overactive H_2_O molecules can lead to severe hydrogen evolution and side reactions (Fig. 2g). In ZHA > 10 electrolytes, the ionic conductivity will be significantly reduced, hindering the conduction of Zn^2+^ (Fig. 2i). In the critical solvation state (ZHA 10), the stable Zn(H_2_O)_6_^2+^ will not be destroyed and due to the existence of the "catcher", active molecules can be arrested during desolvation, promoting the smooth and reversible deposition and stripping of Zn^2+^ (Fig. 2h).

### Critical Solvation Performance Advantage

**Fig. 3:**
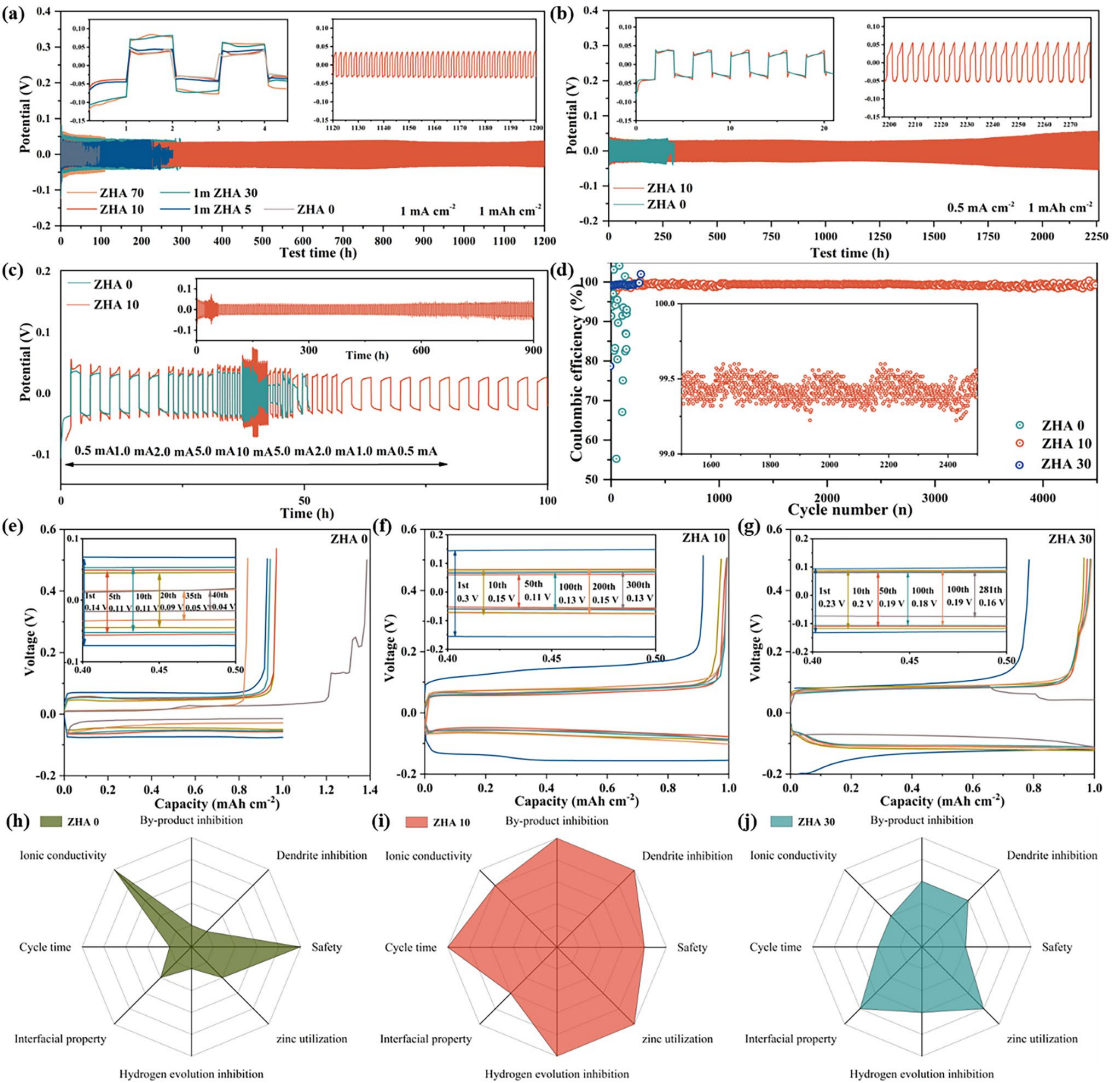
Cycling comparison of symmetric cells in different electrolytes at 1 mAh cm^−2^ and **a** 1 mA cm^−2^, **b** 0.5 mA cm^−2^. **c** Comparison of the rate performance of symmetric cells in ZHA 0 and ZHA 10 electrolytes. **d** Comparison of Coulombic efficiencies of different electrolytes for the Cu||Zn half-cell at a current density of 5 mA cm^−2^. In **e** ZHA 0, **f** ZHA 10, **g** ZHA 30, the voltage polarization curves corresponding to the coulombic efficiency in the electrolyte. Comparison of different properties under **h** ZHA 0, **i** ZHA 10, **j** ZHA 30 solvation structures

In order to demonstrate the excellent performance of ZHA 10 electrolyte, we first tested the cycling performance of Zn||Zn symmetric batteries under different electrolytes at 1 mA cm^−2^ and 1 mAh cm^−2^ (Fig. 3a). Obviously, the performance is best when using ZHA 10 electrolyte, which can maintain a stable cycle without fluctuation for 1,200 h. In contrast, the performance of ZHA 0, ZHA 5, ZHA 30, and ZHA 70 will be significantly worse. Compared with ZHA 0, the overpotential of ZHA 10 did not increase significantly. Based on this, it was verified that the ZHA 10 and ZHA 0 symmetric batteries were compared at 0.5 mA cm^−2^ and 1 mAh cm^−2^ (Fig. 3b), and the ZHA 10 symmetric battery can maintain a stable cycle for 2250 h. ZHA 0 can only be cycled for 270 h before being short-circuited due to dendrites piercing the separator. To further explore its availability at high current densities, a rate test was performed (Fig. 3c). The current density was increased from 0.5 to 10 mA cm^−2^ and then returned to 0.5 mA cm^−2^ for the second time, and the cycle was continued at this current density. Although ZHA 0 can withstand this rate change, when it returns to 0.5 mA cm^−2^ for the next cycle and starts to cycle, it can no longer be maintained due to a short circuit after only 3 cycles. ZHA 10 showed excellent adaptability. After the current density returned to 0.5 mA cm^−2^, it could still maintain a stable cycle without short circuit phenomenon for 900 h. Further embodying the advantages of critical solvation structure.

The coulombic efficiency (CE) of the galvanizing/stripping process was investigated using Cu||Zn half-cells. As shown in Fig. 3d, when the coulombic efficiency is tested at a large current density of 5 mA cm^−2^. It is difficult to maintain a stable coulombic efficiency in both ZHA 0 and ZHA 30 electrolytes. In the ZHA 10 electrolyte, the coulombic efficiency rises rapidly from 95% and then stabilizes at around 99.6%. It can maintain a most stable state between 1000 and 2500 cycles, and even after 10,000 cycles, it can reach a high efficiency of 99.4%. By analyzing the polarization potential, it can be found that the polarization voltage in ZHA 0 electrolyte starts from 0.14 to stable at 0.1 V. However, it can only cycle stably for 30 cycles, and then short circuit occurs due to side reactions and dendrite formation (Fig. 3e). In contrast, in the ZHA 10 electrolyte, the activity of H_2_O molecules is reduced due to the regulation of the hydrogen bond network. The CE of the Zn anode rose rapidly in the first 10 cycles and gradually remained stable, and its polarization voltage decreased from the initial 0.3 to 0.13 V and then remained stable (Fig. 3f). In the ZHA 30 electrolyte, the polarization voltage dropped from 0.23 V to a stable 0.19 V. Due to the increase in the amount of ACN, the decrease in the ionic conductivity of the electrolyte and the reconstitution of the unstable solvation sheath lead to the same short circuit due to dendrites after 300 cycles (Fig. 3g). As shown in Fig. 3h–j, we summarized the cycle time, safety, dendrite inhibition, zinc utilization, ionic conductivity, interface property, hydrogen evolution inhibition, and by-product inhibition capabilities under three different solvation structures. Ultimately, we conclude that critical solvation structures can significantly enhance the reversible electrochemical capabilities of zinc in many ways.

### Critical Solvation Surface Deposition Capability

**Fig. 4:**
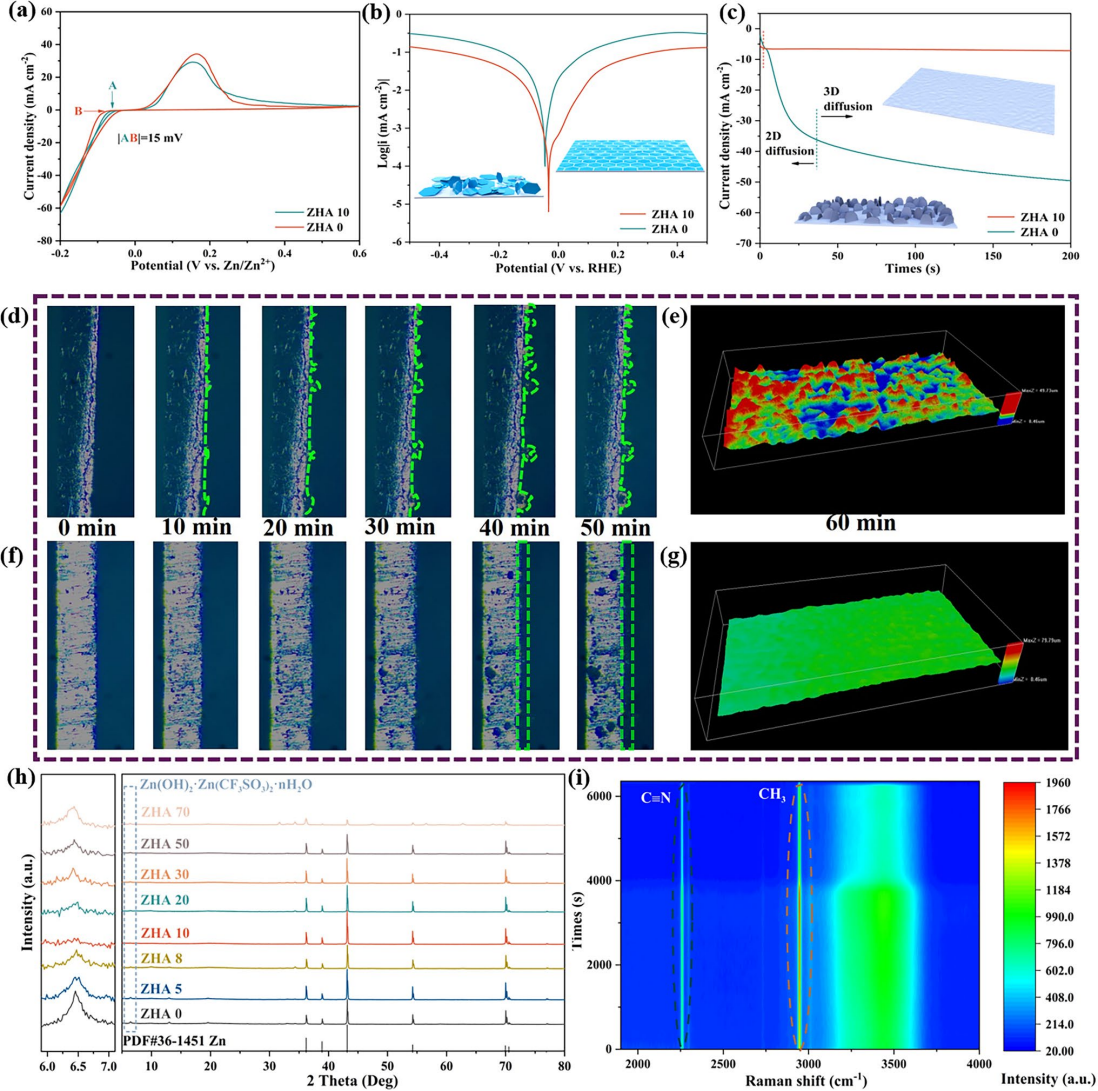
Comparison of **a** CV curve, **b** Tafel curve, **c** CA curve in ZHA 0 and ZHA 10 electrolytes. In **d** ZHA 0 and **f** ZHA 10 electrolytes Zn cross section changes under optical microscope. In **e** ZHA 0 and **g** ZHA 10 electrolytes Zn surface changes under 3D confocal laser scanning microscope. **h** XRD comparison after 100 cycles in different electrolytes. **i** In-situ Raman map of the Zn||Cu battery at 1 mA cm^−2^

The zinc anode will be excessively consumed due to the activity of water in the aqueous electrolyte. We compared the electrochemical stability windows under ZHA 0 and ZHA 10 electrolytes on an inactive titanium (Ti) electrode (Fig. S9). The hydrogen evolution and oxygen evolution potential test of the encapsulated Zn||Ti asymmetric battery. In the ZHA 10 electrolyte, the electrochemical potential window is enlarged (Fig. S9a). Further studies showed that the addition of the “catcher” reduced the hydrogen evolution potential of water from − 40 to − 191 mV (relative to Zn/Zn^2+^) and eliminated the hydrogen evolution current of 0.345 mA cm^−2^ at 25 mV (Fig. S9b). The reason for inhibiting hydrogen evolution is that the “catcher” limits the freedom of water. On the other hand, the oxygen evolution potential was also significantly increased (Fig. S9c). Overall, the limit of reduction potential (− 0.19 V vs. Zn/Zn^2+^) and oxidation potential (2.59 V vs. Zn/Zn^2+^) increases. A wider and more stable electrochemical window of ~ 2.7 V is achieved. CV curves at a scan rate of 1 mV s^−1^ (Fig. 4a). It has been demonstrated that a highly reversible zinc anode is present in ZHA 10 electrolyte, and the initial potential of zinc plating/stripping is better than that in ZHA 0 electrolyte. The effect of ACN additives on the corrosion resistance of zinc anodes was studied by Tafel curves. Theoretically, the corrosion rate of a metal is directly proportional to the corrosion current density, that is, the smaller the corrosion current, the stronger the resistance of the metal to corrosion [23, 59]. As shown in Fig. 4b. The corrosion current measured under ZHA 10 electrolyte is much smaller than that of ZHA 0 electrolyte, indicating that ACN additive significantly inhibits the corrosion of zinc anode. In addition, the CA test curves also indicated extremely short 2D nucleation/growth times in the ZHA 10 electrolyte (Fig. 4c). It shows that more 3D nucleation/growth is going on [60]. As shown in Fig. 4c interior, 2D nucleation leads to uneven Zn deposition, while 3D nucleation promotes uniform deposition growth [61]. The impedance (EIS) spectrum is consistent with the law corresponding to the ionic conductivity (Fig. S10). The above conclusions show that the ZHA 10 electrolyte can make Zn deposition uniform, dense, complete and smooth, inhibit the formation of zinc dendrites, avoid side reactions, and further prolong the service life of zinc anodes.

The galvanization of zinc cross-sections in ZHA 0 and ZHA 10 electrolytes for symmetrical cells was observed using in situ optical microscopy. As shown in Fig. 4d, when ZHA 0 was used, three small tips were formed after 10 min of deposition, and in the subsequent deposition process (0–50 min), the tips became larger and larger, which is the “tip effect”. The 3D confocal laser scanning microscope was used to observe the zinc surface after deposition for 60 min, and it was found that the surface was pitted (Fig. 4e). This deposition becomes dendrites over prolonged cycles and eventually pierces the diaphragm. Correspondingly, as shown in Fig. 4f, when using ZHA 10 electrolyte, the deposition of Zn^2+^ is very flat and smooth, showing that the smaller particles are evenly distributed on the plane. To confirm this view, it can be observed from the 3D depth of field image that the zinc surface is very flat and shiny after 60 min of deposition (Fig. 4g). In addition, we also tested the XRD and SEM changed on the Zn anode surface of the encapsulated symmetric battery after a long cycle in different electrolytes.

As shown in Fig. S11a, after 50 cycles, the basic by-product Zn_x_(CF_3_SO_3_)_y_(OH)_2x−y_·nH_2_O will appear when the proportion of ACN added is small (0, 5%, and 8%). When the addition amount reaches 10%, the generation of by-products is not obvious, until 70%, it appears again. The SEM image can also clearly see that the surface of the zinc anode is smoother when no alkali by-products are produced. When these byproducts were produced, a disordered arrangement of many large, flaky byproducts could be observed (Fig. S11b). For a more detailed comparison, the change of the zinc anode after 100 cycles was tested. It can be observed from Fig. 4h that the basic products of Zn_x_(CF_3_SO_3_)_y_(OH)_2x−y_·nH_2_O are not obvious in ZHA 10 electrolyte, while the basic products of Zn_x_(CF_3_SO_3_)_y_(OH)_2x−y_·nH_2_O are all present in other electrolytes. As shown in Fig. S12, in the ZHA 10 electrolyte, the Zn deposited on the surface of the Zn anode is a reversible white “living zinc”. Therefore, when using the ZHA 10 electrolyte, it can ensure the dense and smooth deposition and stripping of Zn during the cycle, and significantly weaken the generation of zinc dendrites.

In-situ Raman was used to characterize the change of surface electrolyte of Zn anode in Cu||Zn half-cell in ZHA 10 electrolyte. From Figs. 4i and S13, it can be seen that the addition of ACN does not change the deposition and stripping mechanism of Zn^2+^. Figure S14 is the corresponding GCD curve, which shows that only the desolvation process of Zn^2+^ is promoted in ZHA 10 electrolyte. On the other hand, in order to explore whether the battery can form a layer of solid electrolyte interfacial phase (SEI) during cycling. We tested the XPS images of Ar^+^ sputtering of the Zn anode after cycling in the ZHA 0 and ZHA 10 electrolytes for 50 h at 0.5 mA cm^−2^ (Figs. S15–S20) [62]. In the ZHA 0 electrolyte, the Zn surface is more electrolyte residue after cycling. At the same time, it is accompanied by the production of ZnSO_3_, ZnF_2_, ZnS and basic zinc salts. According to previous reports, ZnSO_3_, ZnF_2_, and ZnS can be used as SEI to protect zinc anodes, which is also confirmed by the reported use of Zn(CF_3_SO_3_)_2_ to spontaneously generate SEI. As the etching time increases, this inorganic SEI layer also exists as a protective layer for the zinc anode. In the ZHA 10 electrolyte, the above-mentioned inorganic SEI film can also be perfectly reflected. In addition, it can be observed from Fig. S19 that the N–H bond changes after ACN addition. The hydrogen bonding to water after the addition of ACN was further demonstrated. In addition, from the comparison after etching, the SEI film formed after the addition of ACN is more uniform and flatter. Further, we verified the XPS images of Ar^+^ sputtering after cycling for 100 h (Figs. S21–S26). As the cycle time increases, the SEI film becomes thicker.

By comparing the SEM images of the Zn anode surface and the cross-section (Fig. S27), the Zn anode surface in the ZHA 0 electrolyte obviously has large deposition by-products. The body appears black due to dead zinc (zinc corrosion). In the SEM of the cross section, it was observed that the thickness of the SEI was 0.80 μm, but the whole was uneven. Correspondingly, the zinc anode as a whole still maintains the original white luster of zinc metal after cycling in ZHA 10 electrolyte for 100 h, and the plane observed from the SEM image is very smooth and flat, indicating that the process of electroplating/stripping Zn^2+^ has excellent reversibility, and the zinc deposition is very uniform. The thickness of the SEI film is also only 0.53 μm, significantly thinner than the SEI after cycling in ZHA 0. Therefore, among the dense SEI films formed, the first layer formed on the surface of the Zn anode is an inorganic dense and uniform SEI layer with ZnF_2_ as the main and ZnS as the auxiliary layer, and will continue to form in subsequent deposition. On the surface, ZnCO_3_ and ZnSO_3_ form together to form the SEI component [63]. This SEI layer insulates the electrolyte from direct contact with the zinc anode, and provides a loose porous channel to promote deposition and stripping of Zn^2+^, protecting the zinc anode (Fig. S28). Avoid Zn anode corrosion and passivation caused by direct contact with electrolyte during long-term cycle.

### Critical Solvation Practical Availability

**Fig. 5:**
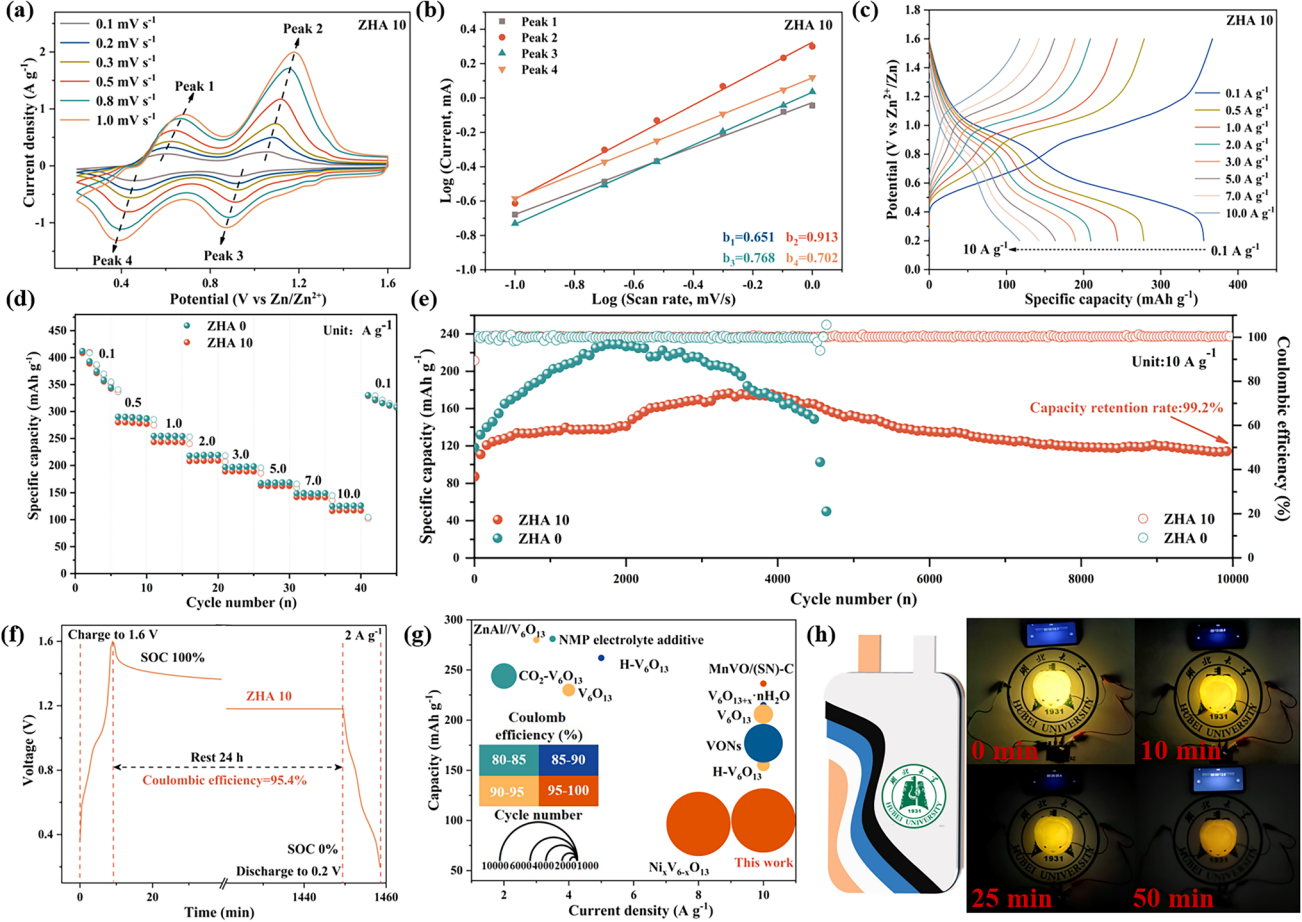
**a** CV curve, **b** fitted b value, **c** GCD curve, **d** ratio performance, **e** long cycle comparison of Zn||V_6_O_13_ battery. **f** Self-discharge in ZHA 10 electrolytes. **g** Comparison of cycling ability of Zn||V_6_O_13_ batteries using ZHA 10 electrolyte with other reported. **h** Soft pack battery lights up the small light

Finally, the feasibility of using ZHA 10 electrolyte in full batteries was verified. We chose V_6_O_13_ as our cathode material. The XRD of V_6_O_13_ powder was tested first. As shown in Fig. S29a, after comparing the PDF card, it can be clearly observed that each diffraction peak of the prepared sample material is basically consistent with the V_6_O_13_ standard comparison card PDF#27-1318. The SEM of Fig. S29b shows that V_6_O_13_ is a layered structure formed by the close packing of nanosheets. Subsequently, the CV curves of the assembled Zn||V_6_O_13_ full cells in ZHA 10 electrolyte were tested. As shown in Fig. 5a, the scanning range is 0.1–1.0 mV s^−1^. The full battery has two redox peaks during the charge–discharge cycle. The corresponding b values were calculated and fitted according to the CV curve, b_1_ = 0.65; b_2_ = 0.91; b_3_ = 0.77; b_4_ = 0.70 (Fig. 5b). The electrochemical kinetics of Zn||V_6_O_13_ battery is dominated by diffusion-controlled process. Figure 5c is the GCD diagram of the full battery. It can be found that the full battery has a specific capacity of 355.7 mAh g^−1^ at 0.1 A g^−1^. It still has a specific capacity of 116.7 mAh g^−1^ at 10 A g^−1^. After adding ACN, the overall redox peak area and the fitted b value of the CV curve (Fig. S30) and the capacity at different current densities (Fig. S31) are lower than those without adding ACN. This is because ACN is an organic solvent, and the addition reduces the ionic conductivity and slows down the diffusion of Zn^2+^ and H^+^.

As shown in Fig. 5d, according to the rate performance, the specific capacity of the ZHA 10 electrolyte at various current densities is lower than that of the ZHA 0 electrolyte, which is consistent with the nature of organic solvents. However, its cycle stability has been greatly improved. The Zn||V_6_O_13_ full battery assembled in ZHA 10 electrolyte is stable for 10,000 cycles at 10 A g^−1^ and has a specific capacity of 100 mAh g^−1^. Compared with the initial stage, the capacity retention rate was 99.2% (Fig. 5e). The self-discharge behavior of the battery is monitored to assess the electrochemical stability of the battery. After charging to 1.6 V, let it stand for 24 h and then discharge to 0.2 V. Compared with using ZHA 0 electrolyte (Fig. S32), using ZHA 10 electrolyte (Fig. 5f) provides a more stable discharge platform and the capacity retention of the battery is stronger. It ensures the excellent corrosion resistance and side reaction resistance of the full battery. Furthermore, the performance of the Zn||V_6_O_13_ full battery using the critical solvation structure was compared with other reported Zn||V_6_O_13_ batteries. As shown in the Fig. 5g, the full cells produced in our work have unparalleled long-term cycling capabilities. The specific parameters of each battery in the Figure are shown in Table S2. To verify the feasibility of Zn||V_6_O_13_ full battery for commercial application, the ZHA 10 electrolyte was used to light up the pouch battery encapsulated with Zn||V_6_O_13_. As shown in Fig. 5h, three Zn||V_6_O_13_ pouch batteries are connected in series to light up the chicken-shaped LED light, which can remain bright after 50 min of lighting. Further proves the feasibility of its commercial application.

## Conclusion

In conclusion, constructing a critical solvation structure electrolyte state can suppress the activity of free water to the greatest extent and reconstruct the hydrogen bond network in the electrolyte. During the deposition process of Zn^2+^, the "catcher" can also arrest the detached active molecules to ensure smooth and reversible electrodeposition of Zn^2+^. The corrosion and hydrogen evolution effect of the zinc anode are inhibited, and the zinc anode is greatly protected. The assembled Zn||Zn symmetric battery can maintain stable cycling for 2250 h at 0.5 mA cm^−2^ and 1 mAh cm^−2^. Zn||V_6_O_13_ full battery can maintain 99.2% capacity of 100 mAh g^−1^ after 10,000 cycles at 10 A g^−1^. The new perspective on the arrest of active molecules by critical solvation structures opens new horizons for designing high-performance reversible zinc electrochemistry.

## Supplementary Information

Below is the link to the electronic supplementary material.

Supplementary file1 (PDF 3019 KB)
