# Supplementary material for: Critical Solvation Structures Arrested Active Molecules for Reversible Zn Electrochemistry
Source: Nanomicro Lett. 2024 Mar 5;16:145. doi: 10.1007/s40820-024-01361-0 (PMC10914662; doi:10.1007/s40820-024-01361-0)
Supplement: Supplementary file 1 — Supplementary file1 (PDF 3019 KB) [file 40820_2024_1361_MOESM1_ESM.pdf]

Supporting Information for

Critical Solvation Structures Arrested Active Molecules for Reversible Zn Electrochemistry

Junjie Zheng[1,2], Bao Zhang[3,*], Xin Chen[1,2], Wenyu Hao[4], Jia Yao[1,2], Jingying Li[1,2], Yi Gan[1,2], Xiaofang Wang[1,2], Xingtai Liu[1,2], Ziang Wu[1,2], Youwei Liu[1,2], Lin Lv[1,2], Li Tao[1,2], Pei Liang[5], Xiao Ji[4], Hao Wang[1,2,*], Houzhao Wan[1,2,*]

[1] Hubei Yangtze Memory Laboratories, Wuhan 430205, P. R. China

[2] Hubei Key Laboratory of Micro-Nanoelectronic Materials and Devices, School of Microelectronics, Hubei University, Wuhan 430062, P. R. China

[3] School of Physical and Mathematical Sciences, Nanyang Technological University, 637371, Singapore, Singapore

[4] School of Optical and Electronic Information, Huazhong University of Science and Technology, Wuhan 430074, P. R. China

[5] Institute of Optoelectronics Technology, China Jiliang University, Hangzhou 310018, P. R. China

* Corresponding authors. E-mail: bao.zhang@ntu.edu.sg (Bao Zhang); nanoguy@126.com, wangh@hubu.edu.cn (H. Wang); houzhaow@hubu.edu.cn (H. Wan)

Supplementary Figures and Tables

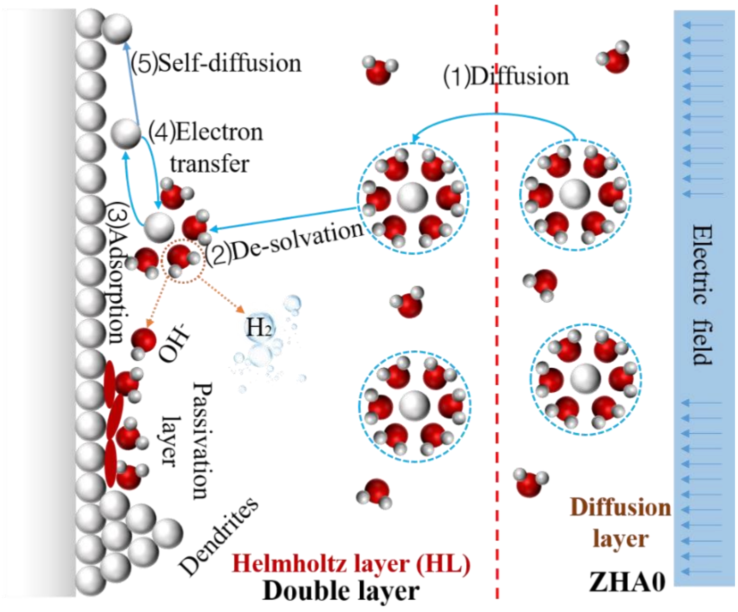

Fig. S1 Electroplating mechanism of Zn^{2+} in ZHA 0 electrolytes

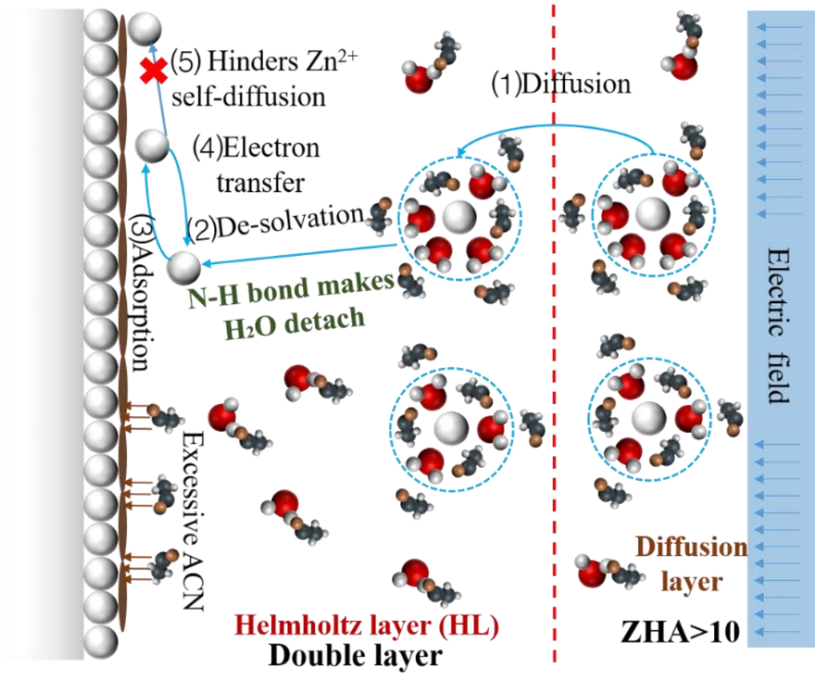

Fig. S2 Electroplating mechanism of Zn^{2+} in ZHA > 10 electrolytes

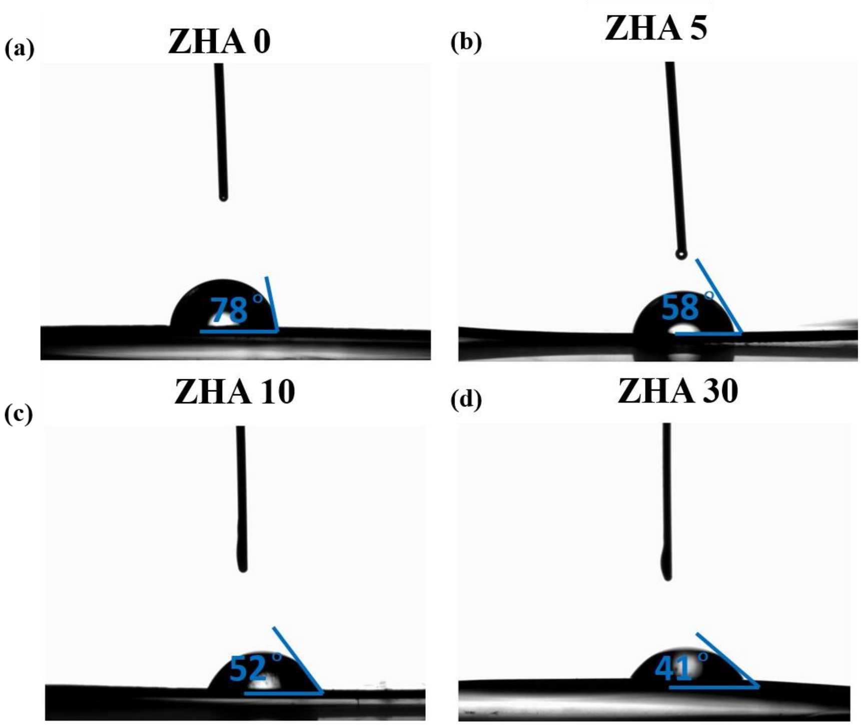

Fig. S3 Contact angles of different electrolytes

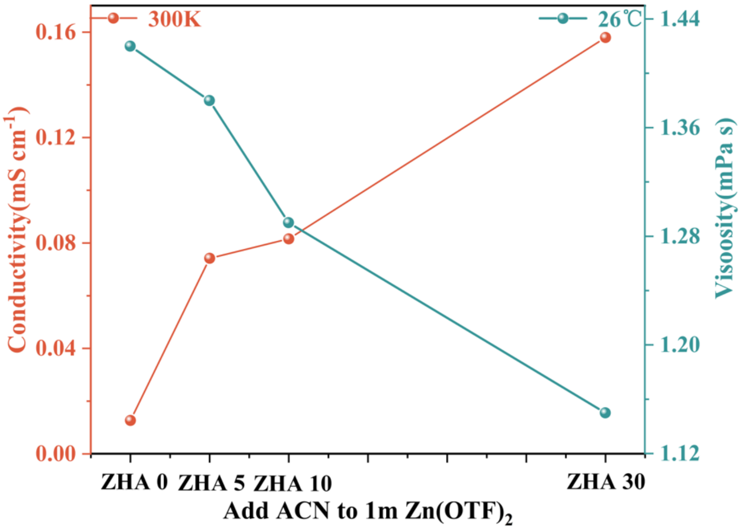

Fig. S4 Ionic conductivity and viscosity of different electrolytes

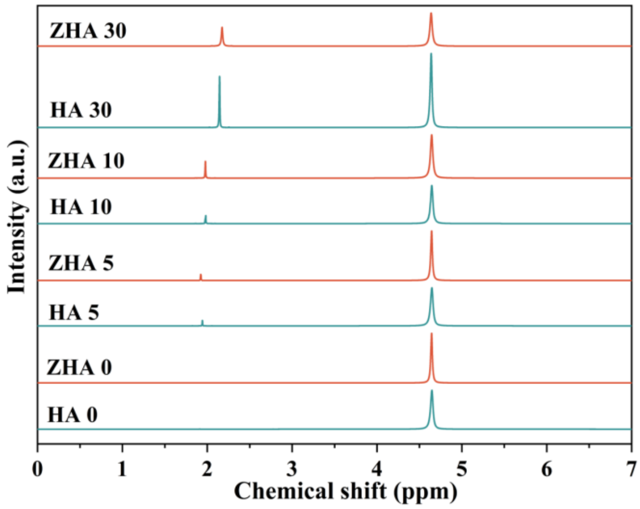

Fig. S5 NMR comparison of different electrolytes

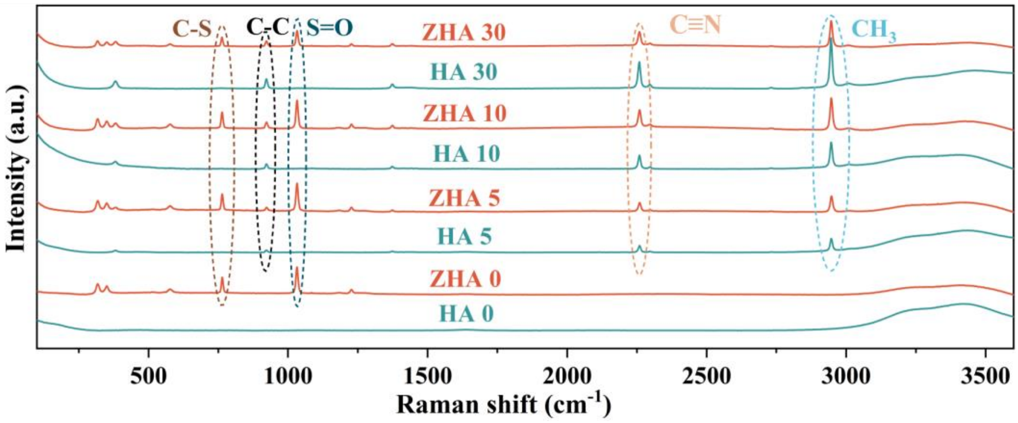

Fig. S6 Raman comparison of different electrolytes

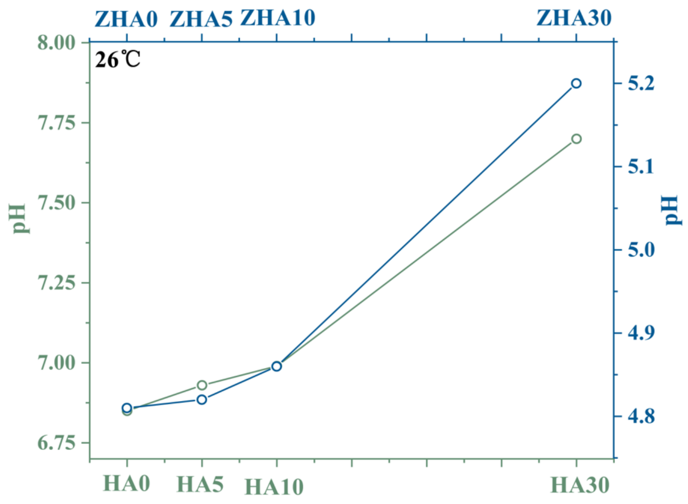

Fig. S7 pH comparison of different electrolytes

	密度（density）	pH	电导率（Conductivity）	粘度（Visoosity）
1m H_2O	1.000	6.85		
1m Zn（OTF）$_2$	1.248	4.81	0.01271	1.42
1m H_2O：ACN=9.5:0.5	0.997	6.93		
1m Zn（OTF）$_2$:ACN=9.5:0.5	1.196	4.82	0.07422	1.38
1m H_2O：ACN=9:1	0.983	6.99		
1m Zn（OTF）$_2$:ACN=9:1	1.146	4.86	0.08157	1.29
1m H_2O：ACN=7:3	0.922	7.89		
1m Zn（OTF）$_2$:ACN=7:3	0.901	5.2	0.158	1.15
pure ACN	0.787			

Fig. S8 Comparison of physical properties of different electrolytes

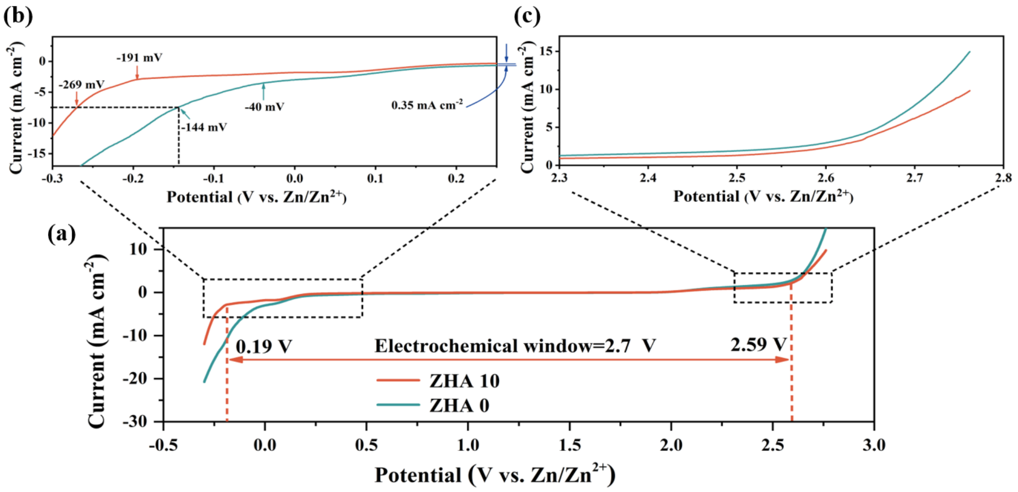

Fig. S9 a Comparison of LSV curves of ZHA 0 and ZHA 10 electrolytes. **b** Hydrogen evolution curve. **c** Oxygen evolution curve

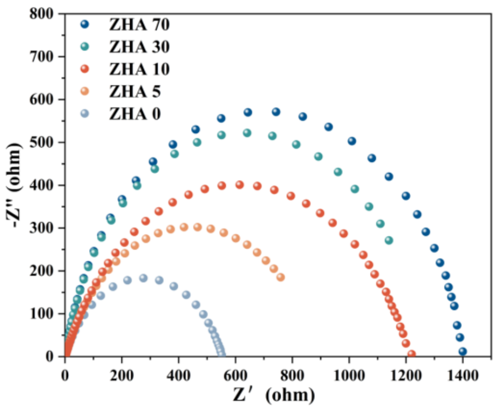

Fig. S10 Electrochemical impedance spectroscopy (EIS) of different electrolytes

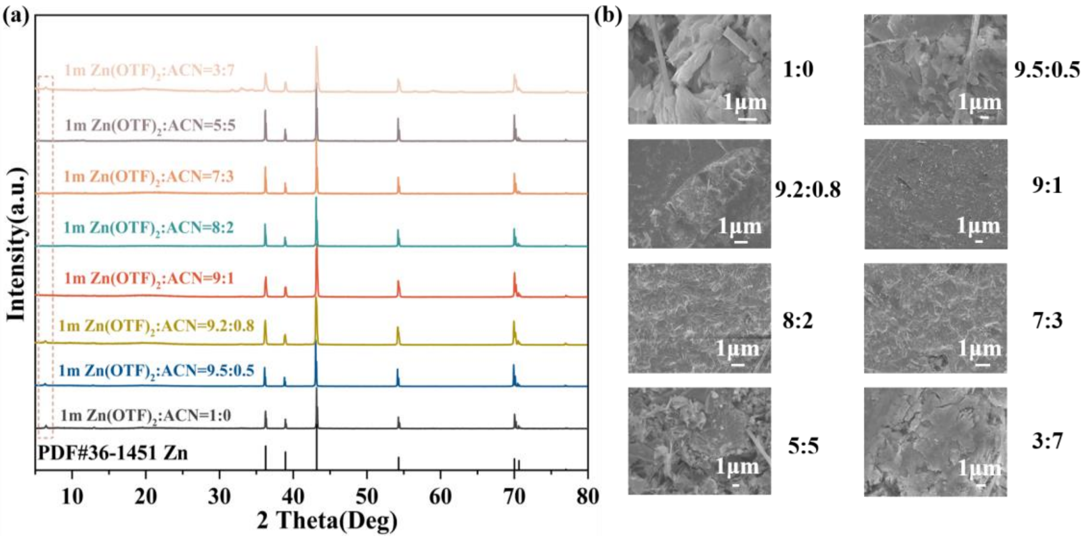

Fig. S11 After 50 cycles of symmetrical cells in different electrolytes **a** XRD image. **b** SEM image

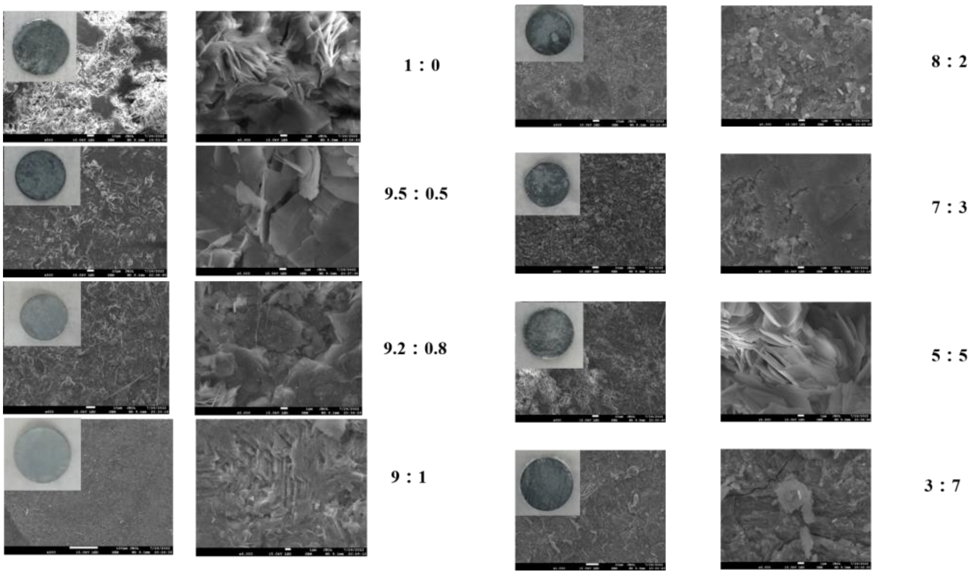

Fig. S12 After 100 cycles of symmetrical cells in different electrolytes SEM image

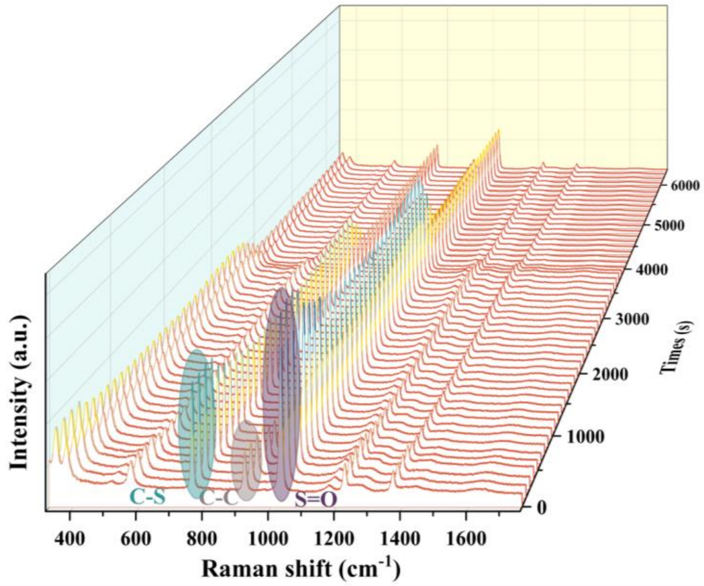

Fig. S13 In-situ Raman map of the Zn||Cu battery at a current density of 1 mA cm^{-2}

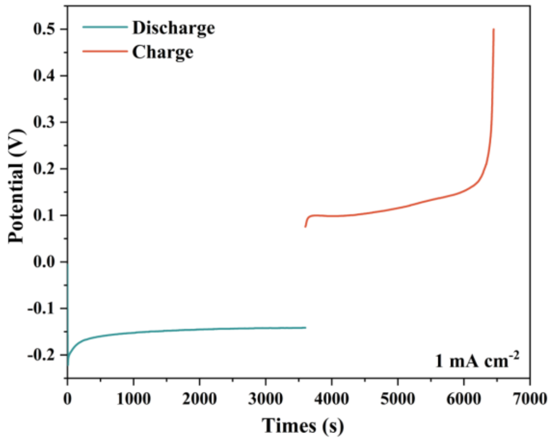

Fig. S14 Zn||Cu half-cell charge-discharge curves corresponding to in-situ Raman

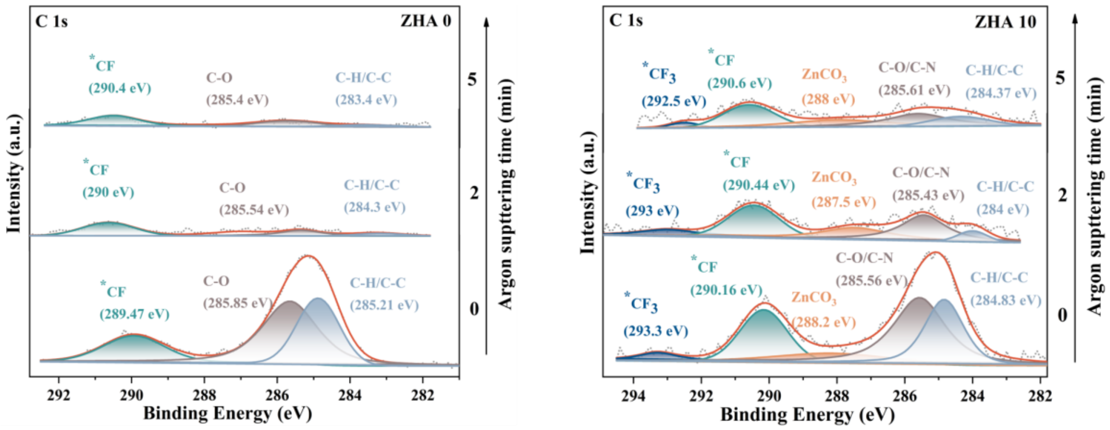

Fig. S15 Elemental C XPS pattern of Ar^{+} sputtered Zn anode after cycling at a current density of 0.5 mA cm^{-2} for 50 h in ZHA 0 and ZHA 10 electrolytes

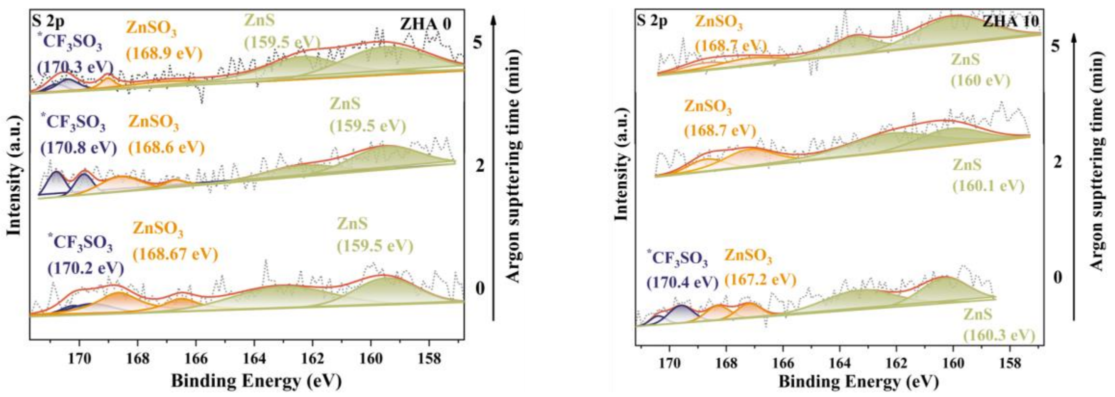

Fig. S16 Elemental S XPS pattern of Ar^{+} sputtered Zn anode after cycling at a current density of 0.5 mA cm^{-2} for 50 h in ZHA 0 and ZHA 10 electrolytes

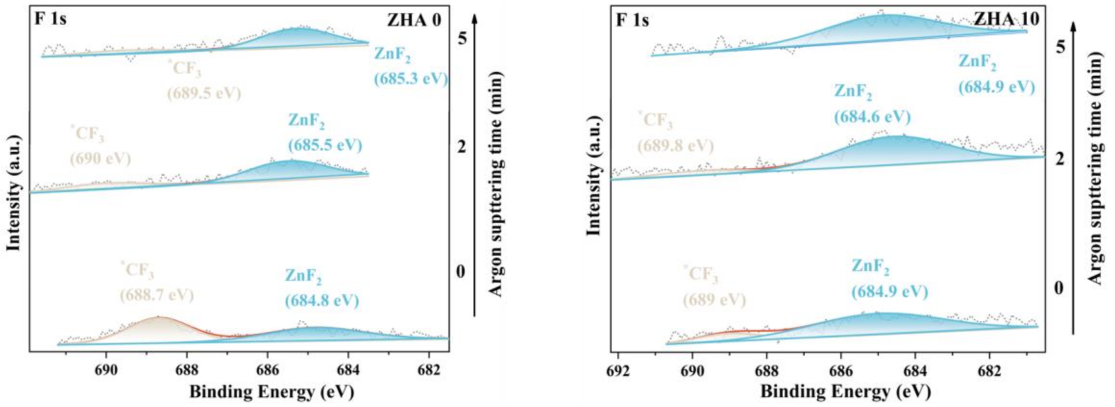

Fig. S17 Elemental F XPS pattern of Ar^{+} sputtered Zn anode after cycling at a current density of 0.5 mA cm^{-2} for 50 h in ZHA 0 and ZHA 10 electrolytes

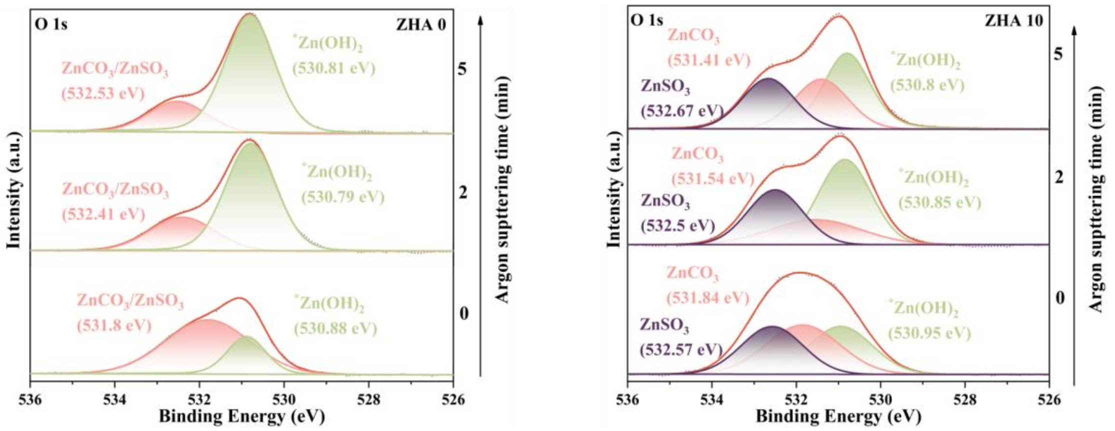

Fig. S18 Elemental O XPS pattern of Ar^{+} sputtered Zn anode after cycling at a current density of 0.5 mA cm^{-2} for 50 h in ZHA 0 and ZHA 10 electrolytes

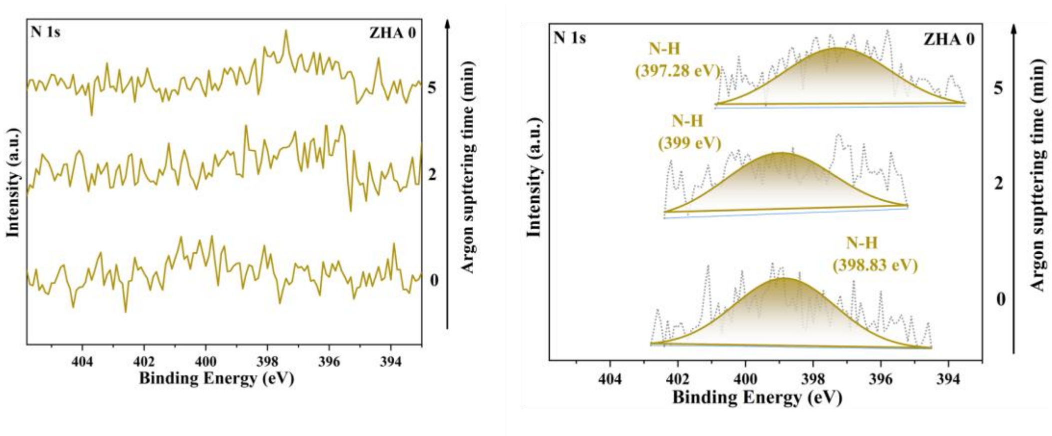

Fig. S19 Elemental N XPS pattern of Ar^+ sputtered Zn anode after cycling at a current density of 0.5 mA cm^{-2} for 50 h in ZHA 0 and ZHA 10 electrolytes

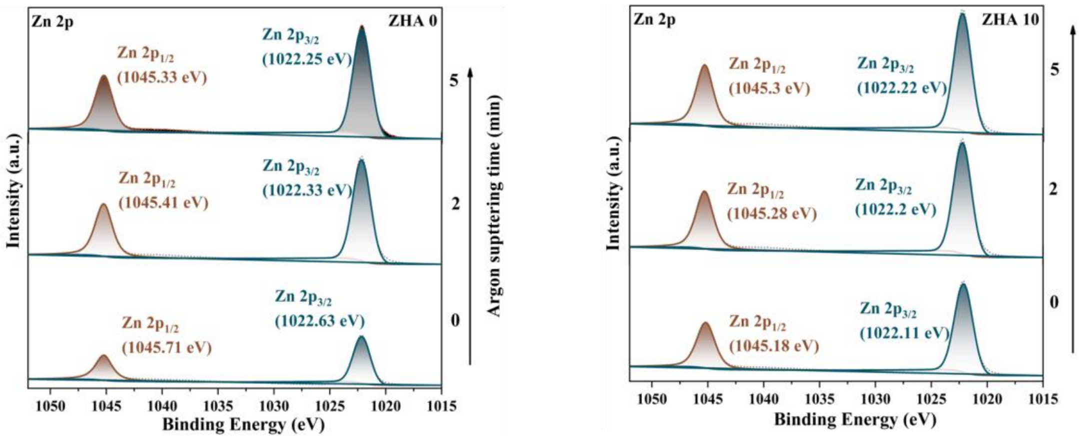

Fig. S20 Elemental Zn XPS pattern of Ar^+ sputtered Zn anode after cycling at a current density of 0.5 mA cm^{-2} for 50 h in ZHA 0 and ZHA 10 electrolytes

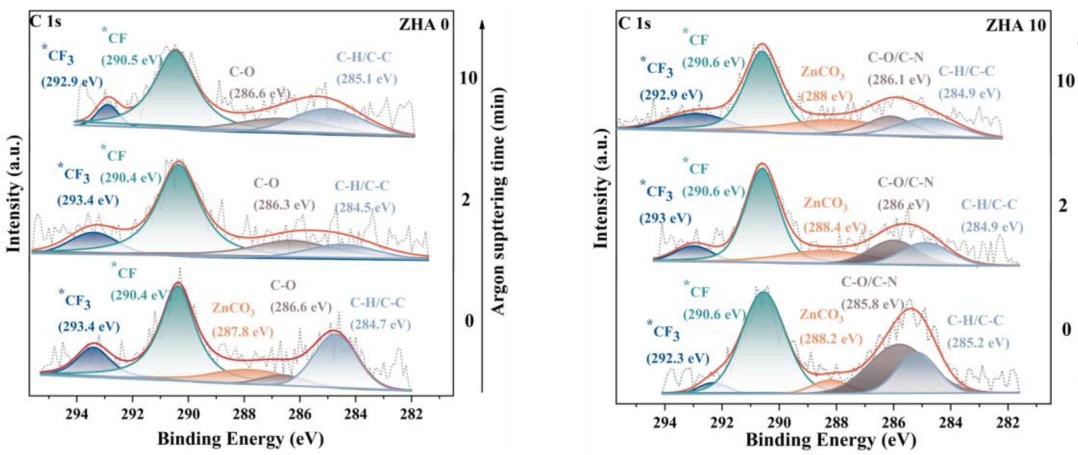

Fig. S21 Elemental C XPS pattern of Ar^+ sputtered Zn anode after cycling at a current density of 0.5 mA cm^{-2} for 100 hours in ZHA 0 and ZHA 10 electrolytes

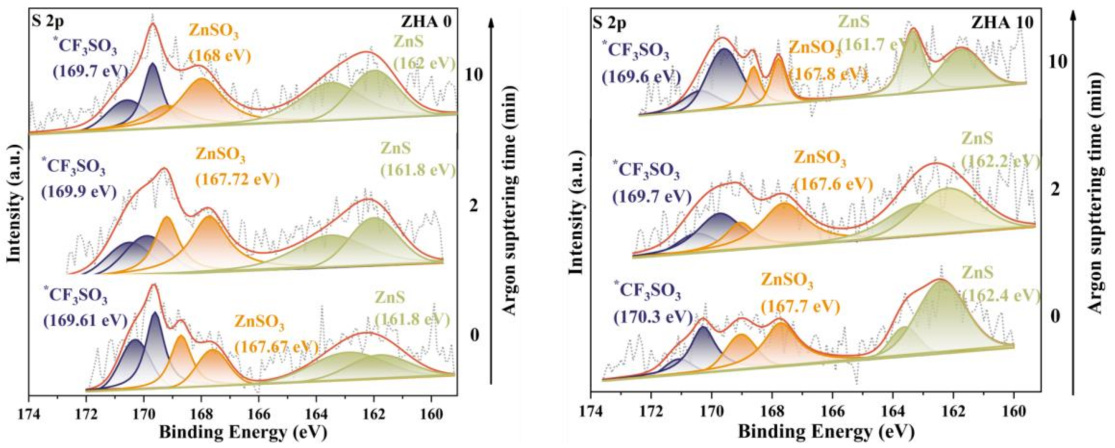

Fig. S22 Elemental S XPS pattern of Ar^+ sputtered Zn anode after cycling at a current density of 0.5 mA cm^{-2} for 100 h in ZHA 0 and ZHA 10 electrolytes

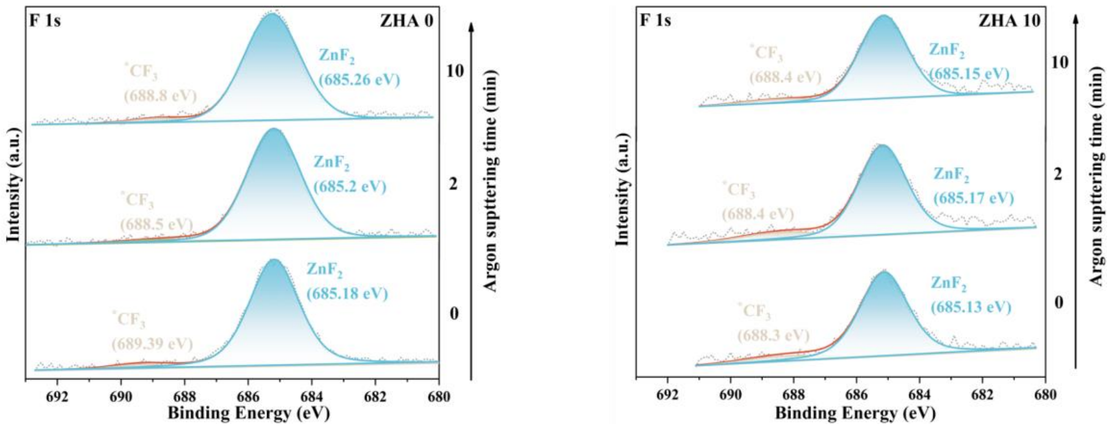

Fig. S23 Elemental F XPS pattern of Ar^+ sputtered Zn anode after cycling at a current density of 0.5 mA cm^{-2} for 100 hours in ZHA 0 and ZHA 10 electrolytes

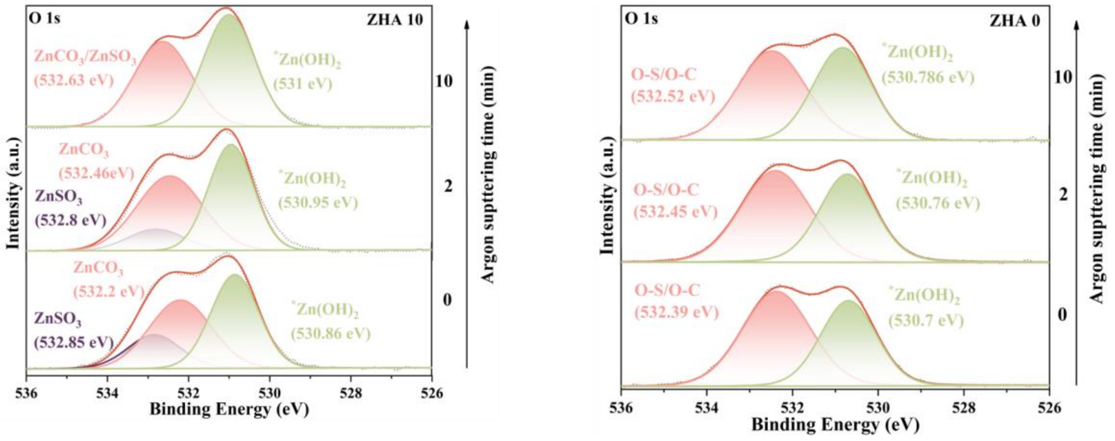

Fig. S24 Elemental O XPS pattern of Ar^+ sputtered Zn anode after cycling at a current density of 0.5 mA cm^{-2} for 100 h in ZHA 0 and ZHA 10 electrolytes

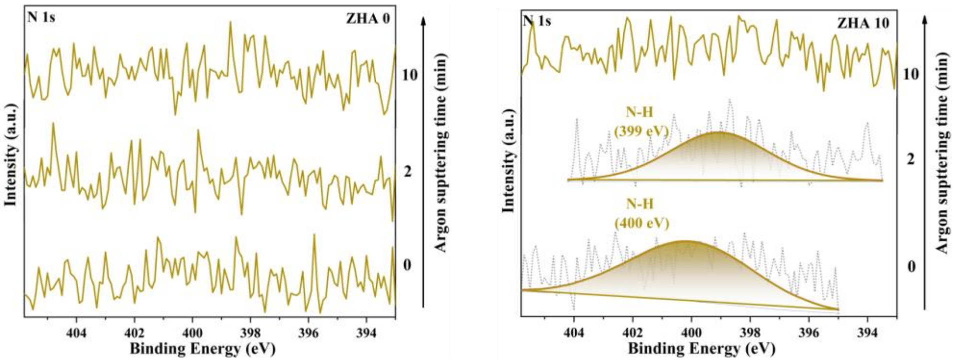

Fig. S25 Elemental N XPS pattern of Ar^+ sputtered Zn anode after cycling at a current density of 0.5 mA cm^{-2} for 100 h in ZHA 0 and ZHA 10 electrolytes

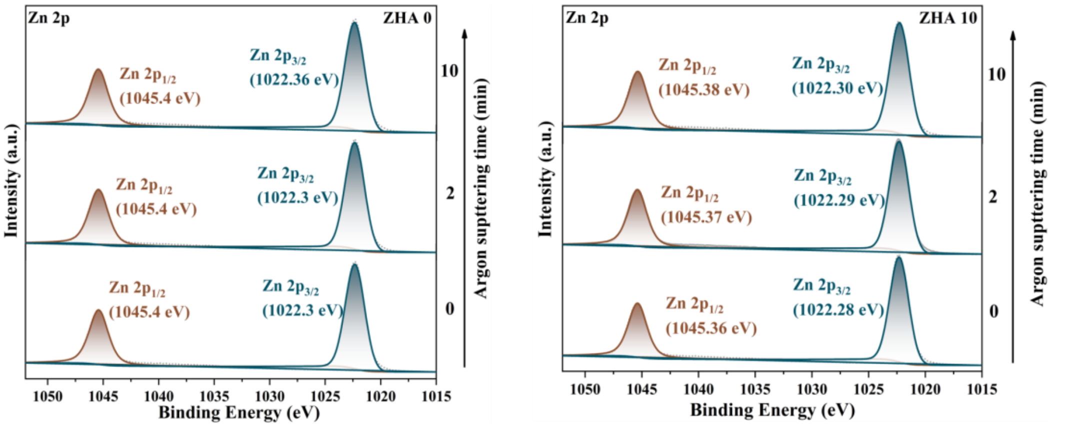

Fig. S26 Elemental Zn XPS pattern of Ar^+ sputtered Zn anode after cycling at a current density of 0.5 mA cm^{-2} for 100 h in ZHA 0 and ZHA 10 electrolytes

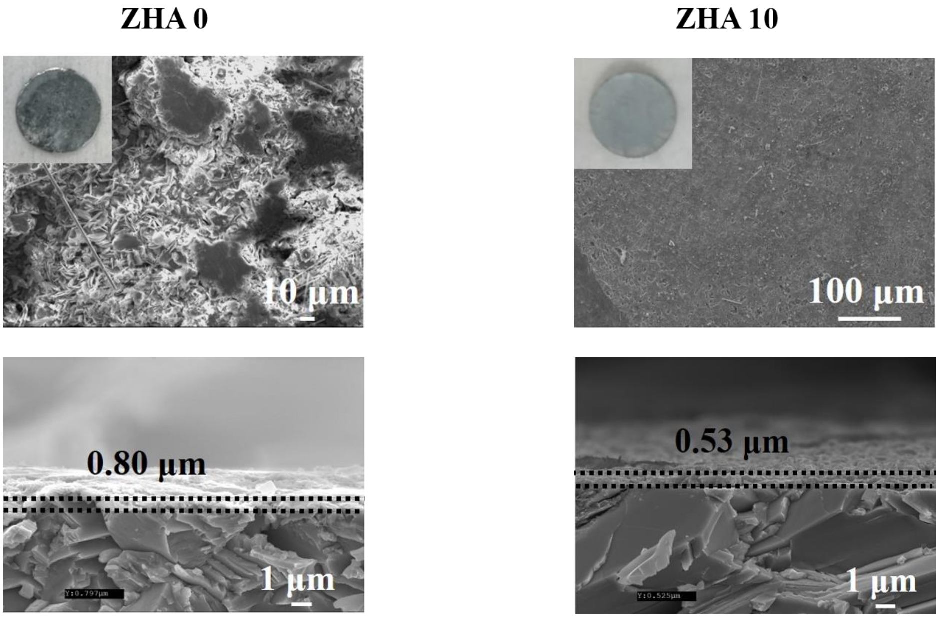

Fig. S27 SEM images of Zn surface and cross-section after cycling the Zn anode in ZHA 0 and ZHA 10 electrolytes at a current density of 0.5 mA cm^{-2} for 100 h

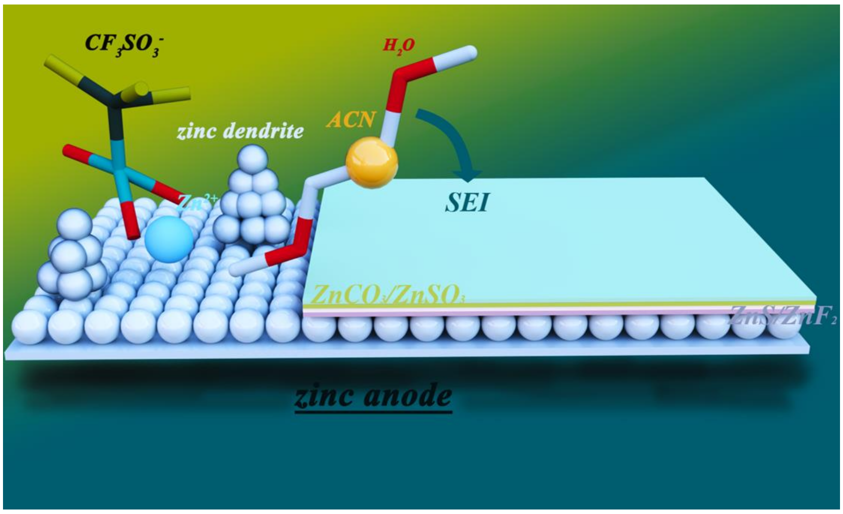

Fig. S28 SEI film formed in ZHA 10 electrolyte

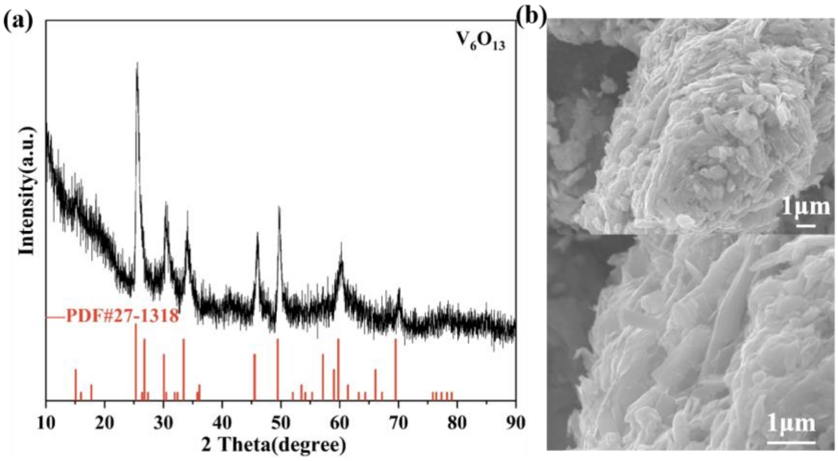

Fig. S29 a XRD and **b** SEM images of V_6O_{13} powder

The formula for analyzing CV data at various scan rates according to a typical method [S1] is as follows:

$i = av^b$

The measured current (i) corresponds to the power-law relationship of the scan rate (v). a and b are tunable parameters, where b is a value determined by the slope of the relationship between log$i$ and log$v$, the coefficient b varies in the range of 0.5–1.0, so there are two clearly defined conditions, namely b = 0.5 and b = 1.0. The b value is 0.5, which indicates the insertion process of diffusion control, and the b value is 1.0, which indicates the surface capacitance process. Linear relationship between log i and log v graphs (log$i$ = log$a$ + $b$log$v$) according to **Fig. S28** [S2]. The b values of the four redox peaks are calculated to be 0.68 (peak 1), 0.92 (peak 2), 0.89 (peak 3) and 0.83 (peak 4), This indicates that the electrochemical kinetics of the compounds are related to diffusion-controlled processes and capacitive effects, but the surface capacitance process are the main process.

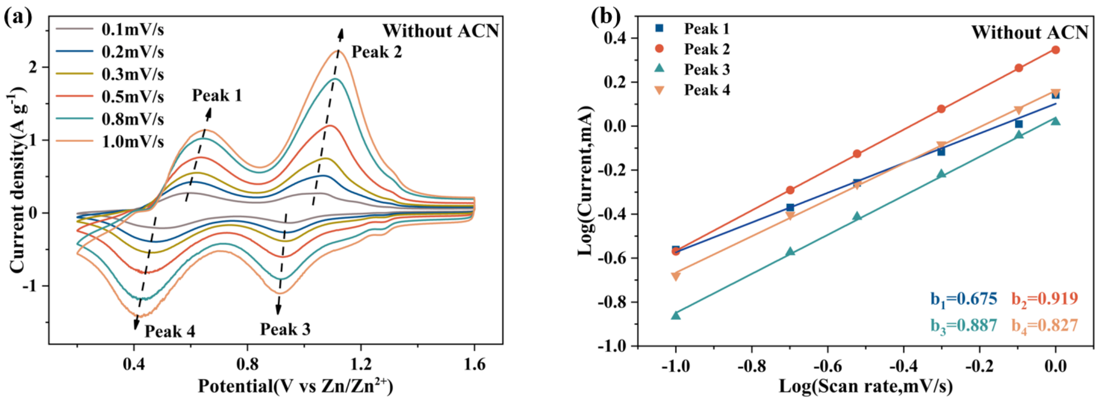

Fig. S30 a CV plots and **b** b-values of Zn||V_6O_{13} full cells in ZHA 0 electrolyte

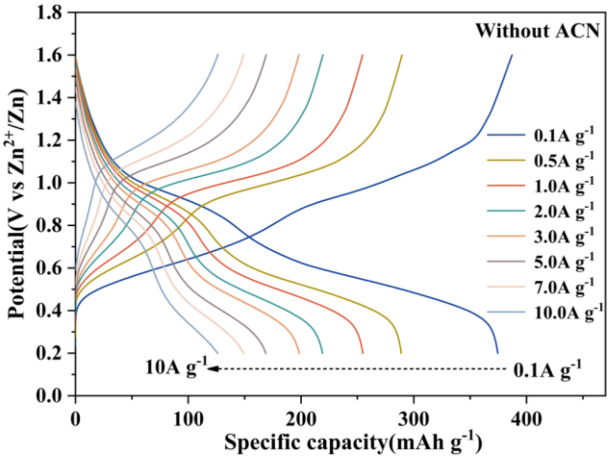

Fig. S31 GCD plots of Zn||V_6O_{13} full cells in ZHA 0 electrolyte

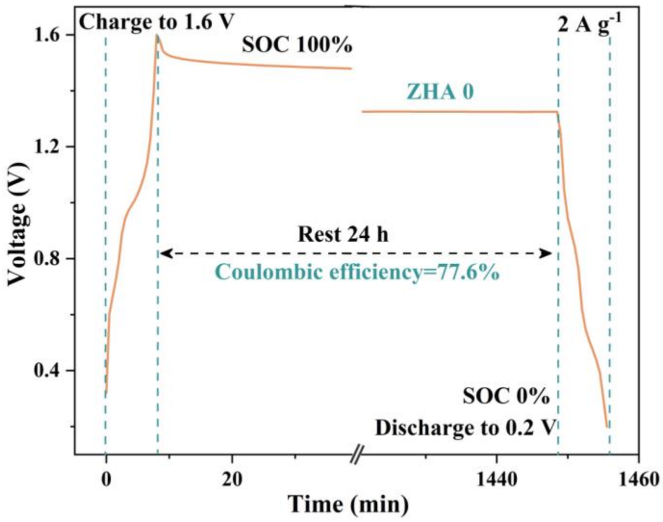

Fig. S32 Zn||V_6O_{13} self-discharge in ZHA 0 electrolytes

Table 1 Coordination number of acetonitrile at different proportions

	H_2O	Otf^-	ACN
0% ACN	5.536	0.464	0
5% ACN	5.719	0.281	0
10% ACN	5.754	0.246	0
20% ACN	5.571	0.388	0.04
30% ACN	5.56	0.415	0.05

Table 2 Performance comparison table of Zn||V_6O_{13} batteries reported in this work and other reports

**Description**	**Current density ($A\ g^{-1}$)**	**Cycle number**	**Capacity ($mAh\ g^{-1}$)**	**Coulomb efficiency (%)**	**References**
H-V_6O_{13}	5	1000	262	87	[S3]
CO_2-V_6O_{13}	2	4000	244	80	[S4]
$V_6O_{13+x}\cdot nH_2O$	10	1000	215	89	[S5]
K-V_6O_{13}	10	2000	155.5	90.8	[S6]
V_6O_{13}	10	3000	206	91.5	[S7]
ZnAl//V_6O_{13}	3	1000	280	95	[S8]
$Ni_xV_{6-x}O_{13}$	8	10000	96.5	99	[S9]
V_6O_{13}	4	2000	230	92	[S10]
MnVO/(SN)-C	10	1000	236.4	99	[S11]
NMP eletrolyte additive	3.5	1000	281	82.4	[S12]
VONs	10	6000	177	88.9	[S13]
**This work**	**10**	**10000**	**100**	**99.2**	

Supplementary References

[S1] V. Augustyn, J. Come, M.A. Lowe, J.W. Kim, P.-L. Taberna et al., High-rate electrochemical energy storage through Li^+ intercalation pseudocapacitance. Nat. Mater. **12**(6), 518–522 (2013). https://doi.org/10.1038/nmat3601

[S2] T. Brezesinski, J. Wang, J. Polleux, B. Dunn, S.H. Tolbert, Templated nanocrystal-based porous TiO_2 films for next-generation electrochemical capacitors. J. Am. Chem. Soc. **131**(5), 1802–1809 (2009). https://doi.org/10.1021/ja8057309

[S3] J. Lai, H. Zhu, X. Zhu, H. Koritala, Y. Wang, Interlayer-expanded $V_6O_{13}\cdot nH_2O$ architecture constructed for an advanced rechargeable aqueous zinc-ion battery. ACS Appl. Energy Mater. **2**(3), 1988–1996 (2019). https://doi.org/10.1021/acsaem.8b02054

[S4] W. Shi, B. Yin, Y. Yang, M.B. Sullivan, J. Wang et al., Unravelling V_6O_{13} diffusion pathways via CO_2 modification for high-performance zinc ion battery cathode. ACS Nano **15**(1), 1273–1281 (2021). https://doi.org/10.1021/acsnano.0c08432

[S5] B. Sambandam, S. Kim, D.T. Pham, V. Mathew, J. Lee et al., Hyper oxidized $V_6O_{13+x}\cdot nH_2O$ layered cathode for aqueous rechargeable Zn battery: Effect on dual carriers transportation and parasitic reactions. Energy Storage Mater. **35**(47–61), (2021). https://doi.org/10.1016/j.ensm.2020.11.001

[S6] Q. Chen, Z. Luo, X. Zhao. K-ion intercalated V_6O_{13} with advanced high-rate long-cycle performance as cathode for zn-ion batteries. J. Mater. Chem. C **10**(2), 590–597 (2022). https://doi.org/10.1039/D1TC04822H

[S7] L. Shan, J. Zhou, W. Zhang, C. Xia, S. Guo et al., Highly reversible phase transition endows V_6O_{13} with enhanced performance as aqueous zinc-ion battery cathode. Energy Technol. **7**(6), 1900022 (2019). https://doi.org/10.1002/ente.201900022

[S8] Z. Qi, T. Xiong, Z.G. Yu, F. Meng, B. Chen et al., Suppressing zinc dendrite growth in aqueous battery via Zn–Al alloying with spatially confined zinc reservoirs. J. Power Sources **558**, 232628 (2023). https://doi.org/10.1016/j.jpowsour.2023.232628

[S9] Y.-Y. Liu, G.-Q. Yuan, X.-Y. Wang, J.-P. Liu, Q.-Y. Zeng et al., Tuning electronic structure of ultrathin V_6O_{13} nanobelts via nickel doping for aqueous zinc-ion battery cathodes. Chem. Eng. J. **428**, 132538 (2022). https://doi.org/10.1016/j.cej.2021.132538

[S10] J. Shin, D.S. Choi, H.J. Lee, Y. Jung, J.W. Choi, Hydrated intercalation for high-performance aqueous zinc ion batteries. Adv. Energy Mater. **9**(14), 1900083 (2019). https://doi.org/10.1002/aenm.201900083

[S11] X. Wang, L. Ye, Y. Zou, L. Zhao, Q. Jiang, Constructing ultra-long life and super-rate rechargeable aqueous zinc-ion batteries by integrating mn doped V_6O_{13} nanoribbons with sulfur-nitrogen modified porous carbon. Mater. Today Energy **19**, 100593 (2021). https://doi.org/10.1016/j.mtener.2020.100593

[S12] K. Wang, F. Liu, Q. Li, J. Zhu, T. Qiu et al., An electrolyte additive for interface regulations of both anode and cathode for aqueous zinc-vanadium oxide batteries. Chem. Eng. J. **452**, 139577 (2023). https://doi.org/10.1016/j.cej.2022.139577

[S13] M. Chen, H. Kimura, X. Yang, H. Huang, C. Hou et al., Carbon reduction assist structured preparation stable three-dimensional V_6O_{13} for durable aqueous zinc-ion batteries. J. Power Sources **580**, 233332 (2023). https://doi.org/10.1016/j.jpowsour.2023.233332
